# Comparison of basal ganglia regions across murine brain atlases using metadata models and the Waxholm Space

**DOI:** 10.1038/s41597-024-03863-3

**Published:** 2024-09-27

**Authors:** H. Kleven, U. Schlegel, H. J. Groenewegen, T. B. Leergaard, I. E. Bjerke

**Affiliations:** 1 Neural Systems Laboratory, Institute of Basic Medical Sciences, University of Oslo, Oslo, Norway; 2 Department of Anatomy and Neurosciences, Amsterdam University Medical Center, Amsterdam, The Netherlands

**Keywords:** Computational neuroscience, Neuroscience

## Abstract

The murine basal ganglia regions are targets for research into complex brain functions such as motor control and habit formation. However, there are several ways to name and annotate these regions, posing challenges for interpretation and comparison of data across studies. Here, we give an overview of basal ganglia terms and boundaries in the literature and reference atlases, and describe the criteria used for annotating these regions in the Waxholm Space rat brain atlas. We go on to compare basal ganglia annotations in stereotaxic rat brain atlases and the Allen Mouse brain Common Coordinate Framework to those in the Waxholm Space rat brain atlas. We demonstrate and describe considerable differences in the terms and boundaries of most basal ganglia regions across atlases and their versions. We also register information about atlases and regions in the openMINDS metadata framework, facilitating integration of data in neuroscience databases. The comparisons of terms and boundaries across rat and mouse atlases support analysis and interpretation of existing and new data from the basal ganglia.

## Introduction

The term “basal ganglia” is a conceptual grouping of several deeply situated brain regions for which anatomical boundaries have been subject to debate. These regions have long attracted attention for their involvement in important brain functions such as motivational control of movement, habit formation, and reward processing^1–4^. Based on histological and chemoarchitectural characteristics, axonal connections and functional properties, the basal ganglia are generally recognised as a set of regions which from rostral to caudal include the striatum, pallidum, subthalamic nucleus and nigral complex^5^. The striatum consists of the caudate-putamen and nucleus accumbens, receives axonal projections from the cerebral cortex, and forms the main point of input to the basal ganglia. The pallidum consists of an internal and external segment, as well as the ventral pallidum. The nigral complex consists of a reticular and a dopaminergic part. The internal pallidal segment, ventral pallidum, and reticular part of the substantia nigra constitute the output part of the basal ganglia, giving rise to axonal projections directed primarily via the thalamus to the cerebral cortex. The other mentioned subdivisions form an intricate intra-basal ganglia circuit that interconnects virtually all parts of the basal ganglia^6^.

The detailed neuroanatomical organisation of the basal ganglia has long been subject to debate^7,8^. Recently, the first detailed, three-dimensional (3D) representation of the rat basal ganglia was made available through version 4 of the *Waxholm Space atlas of the Sprague Dawley rat brain* (*WHS rat brain atlas v4*)^9^. The practical value of this atlas for integration of data and brain-wide analyses has been emphasised in our previous publication^9^. However, this atlas adds to a landscape of substantial variability in region terms and boundaries across the literature and other brain atlases^10–13^. It is difficult to get an overview of commonly used terms and how they differ from each other, or to reconcile findings reported using different brain atlases or custom boundaries, let alone to compare data across species.

To address these gaps in our knowledge, we have investigated how the basal ganglia terms and boundaries vary across literature and atlases for the rat and mouse brain. We first briefly describe the main regions of the basal ganglia and the most prominent debates about naming conventions. We go on to provide a detailed description of the boundaries made when delineating the basal ganglia regions in the *WHS rat brain atlas v4*. We furthermore assess how the basal ganglia regions in two commonly used rat brain atlases spatially relate to the *WHS rat brain atlas v4*. Such an overview is an important basis for interpreting findings and for making comparisons to information reported in the literature. We organise all the rat brain atlases surveyed here and integrate them into the openMINDS metadata framework (https://github.com/openMetadataInitiative/openMINDS) as standardised machine-readable resources, facilitating their use in graph databases and research. Lastly, to support cross-species comparisons, we also investigate the spatial correspondence between basal ganglia regions in the *WHS rat brain atlas v4* and the *Allen CCFv3 2017*^14^. Together, our comparisons provide a basis for understanding basal ganglia terms and definitions across rat and mouse brain atlases, paving the way for more transparent reporting and reconciliation of findings from these brain regions.

## Results

To compare and integrate data referencing different brain atlases and their versions, we need to understand how the terms and boundaries of the basal ganglia vary across them. In the following, we first give a brief overview of the basal ganglia regions and review the terms, subdivision and hierarchical organisation schemas that prevail in the literature. We then present the terms and boundaries of the basal ganglia regions in the *Waxholm Space atlas of the Sprague Dawley rat brain version 4* (*WHS rat brain atlas v4*). We go on to survey the basal ganglia in *The Rat Brain in Stereotaxic Coordinates* by Paxinos and Watson^10,12,15–19^ and *Brain Maps: Structure of the Rat Brain* by Swanson^11,13,20,21^, reviewing changes in the nomenclature and annotations across versions. We then analyse how these annotations relate to regions in the *WHS rat brain atlas v4*. Lastly, to provide a basis for cross-species comparison studies, we compare the basal ganglia regions in the *WHS rat brain atlas v4* to those found in the *Allen Mouse Brain atlas Common Coordinate Framework version 3, 2017*.

### A brief overview of basal ganglia regions, hierarchy, and nomenclature

**Fig. 1:**
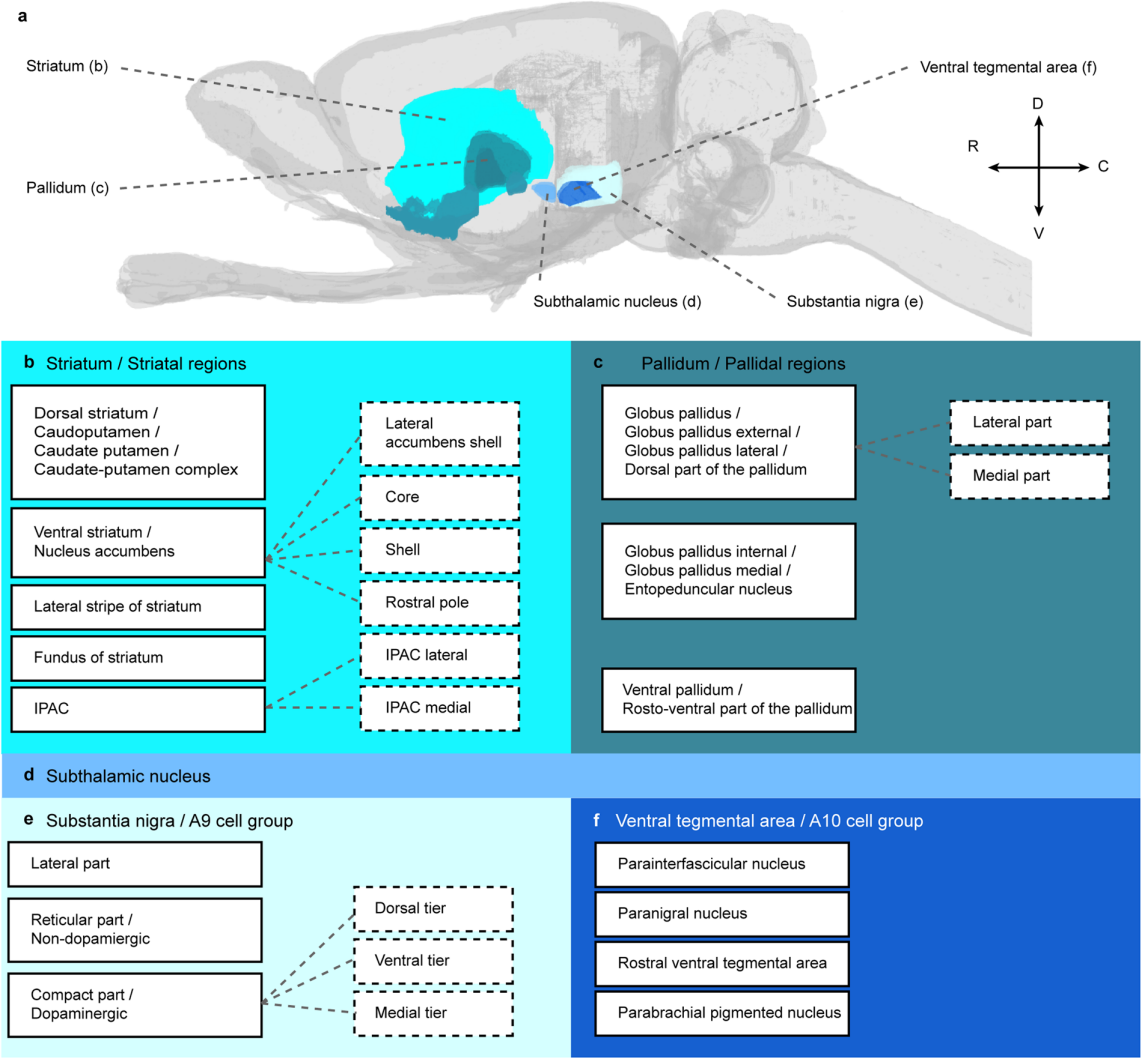
Basal ganglia terms in murine brain atlases. (**a**) Lateral view of a transparent rat brain, with basal ganglia regions in colour. The basal ganglia consist of striatum, pallidum, subthalamic nucleus, substantia nigra and ventral tegmental area. The most common region and subregion terms for the striatum (**b**), pallidum (**c**), subthalamic nucleus (**d**), substantia nigra (**e**) and ventral tegmental area (**f**) are provided. Boxes with solid lines are regions and those with dashed lines are subregions. Note that for all these regions, only some lexical variants of a term are represented here. **Abbreviations:** C, caudal; D, dorsal; IPAC, interstitial nucleus of the posterior limb of the anterior commissure; R, rostral; V, ventral.

The basal ganglia consist of striatum, pallidum, subthalamic nucleus, ventral tegmental area, and substantia nigra (Fig. 1a). Technically, the term “ganglia” is intended to describe cell groups in the peripheral nervous system and the anatomically correct term for the basal ganglia would therefore be basal nuclei^22^. Due to the long tradition of the term “basal ganglia” across literature and atlases, we nevertheless choose to use this term in the current work. The term basal ganglia was originally reserved for the large subnuclear masses in the (human) forebrain, also termed the corpus striatum^22^, which included the caudate nucleus and the lentiform nucleus. The latter, located lateral to the internal capsule, included the putamen laterally and the globus pallidus medially. Subsequently, it was recognised that the caudate nucleus and putamen have similar histological and hodological characteristics and should be considered as one nuclear mass, i.e., the striatum, be it that they are spatially separated by the internal capsule. With the advances of neuroanatomical techniques to describe the neuronal connections within the brain, it became clear how intricately the subthalamic nucleus and the substantia nigra/VTA-complex, located respectively in the diencephalon and mesencephalon, are connected with these basal nuclei in the forebrain. Therefore, it is now generally accepted to include all of the above-mentioned structures into the ‘basal ganglia’. The basal ganglia are thus rather a functional than a morphological entity. In the following, we briefly describe the most common terms and subdivisions for the basal ganglia regions in rat and mouse (collectively referred to as murine) atlases and in the literature (Fig. 1b–f).

The striatum consists of the caudate-putamen and nucleus accumbens (Fig. 1b), both of which receive extensive axonal projections from across the cortical mantle and from thalamic regions^23–26^. Thus, the striatum is an input region of the basal ganglia and the origin of the direct and indirect pathways through the system^1,6^. In the primate, the caudate and the putamen are separated into two clearly distinguishable regions by the internal capsule; however, these fibres are distributed as scattered fibre bundles in the rodent, and a clear boundary between the caudate and putamen is not present (hence the term “caudate-putamen”). Because of the relative anatomical positions of caudate-putamen and nucleus accumbens, they are often referred to as the dorsal and ventral striatum, respectively. Both sets of terms (“caudate-putamen” and “nucleus accumbens” or “dorsal striatum” and “ventral striatum”) are widely used. As both the caudate-putamen and nucleus accumbens consist of mainly medium spiny projection neurons^5^ relatively homogeneously distributed across the two regions, the border between them is hard to define. It should be noted that the term “striatum” in the murine literature is commonly used to refer exclusively to the caudate-putamen. There are substantial variations in the neural connections and functional properties of the striatum along the dorsolateral-to-ventromedial and rostrocaudal axes^24,27,28^. The nucleus accumbens represents the most ventromedial part of the striatum, and is often divided into a core and shell^27,29,30^. Some consider the rostral-most part of the nucleus accumbens shell to be a separate entity with distinct efferent projection patterns, indicated as the rostral pole^30,31^. While the core and shell have been the most widely recognised subregions, it is also acknowledged that each subregion shows further complex internal organisation into functionally and hodologically defined subterritories or neuronal ensembles^32^. For example, the caudomedial shell stands out as a rather distinct area and as such constitutes a zone of transition between striatal and extended amygdalar areas^8,33^. Interestingly, there are indications that the caudal end of the striatum (which is exclusively occupied by the caudate-putamen) represents a subterritory with distinct anatomical and functional properties^28,34^. Despite the knowledge of subterritories in the caudate-putamen and nucleus accumbens core and shell^35^, clear-cut borders based on histological or immunohistochemical criteria are not available; as a result, such subterritories are seldom represented in atlases.

For the murine pallidum, there have been extensive debates regarding subdivisions, naming conventions, and possible homologies with primate species (Fig. 1c). As for the striatum, the concepts of the pallidal regions were originally established in primates, where the globus pallidus consists of immediately adjacent, clearly distinguishable internal (medial) and external (lateral) parts. The internal part is one of the main output nuclei of the basal ganglia, relaying striatal signals to its output regions through the so-called direct pathway. The external part receives input from the striatum and sends efferent projections to the subthalamic nucleus, forming part of the indirect pathway through the basal ganglia^6^. As in the primate, the murine brain has a pallidal area located directly medially to the caudate-putamen and laterally to the internal capsule. This area has a large population of parvalbumin-positive neurons and is heavily connected to the subthalamic nucleus^36,37^. It has long been believed that this area is homologous to only the external or lateral part of the primate globus pallidus, and it has usually been referred to as the external globus pallidus or simply globus pallidus in murine atlases and literature^10,11,13,14,19,38,39^ (in the following, we refer to it as “globus pallidus”). The murine entopeduncular nucleus, embedded in the internal capsule closer to the hypothalamus, has been widely believed to represent the homologue of the primate internal globus pallidus, based on the highly similar connectivity of these regions^5^. The terms “internal” and “external” are often exchanged for “medial” and “lateral” when denoting the pallidal regions. The terms “entopeduncular nucleus” and “internal globus pallidus” have often been interchangeably used in the murine literature, and both appear in different murine brain atlases. However, Puelles and colleagues^40^ recently summarised the historical notions and recent experimental evidence regarding the developmental, molecular and hodological identity of different parts of the murine pallidum, arguing against an internal globus pallidus-entopeduncular nucleus homology. They argue that the murine globus pallidus (often referred to as the external or lateral globus pallidus) is homologous with both the internal and external segments of the primate globus pallidus. There is indeed evidence pointing towards a dichotomous organisation of the murine globus pallidus, based on molecular, anatomical, electrophysiological and hodological criteria^41–43^. Puelles and colleagues further posit that the murine entopeduncular nucleus is of hypothalamic origin and consists of several distinct subregions, for which the primate homologues are currently unknown^40^. Thus, novel data now seem to change the notion of the murine entopeduncular nucleus as homologous to the primate internal globus pallidus.

It has long been noted that there is a rostro-ventral extension to the murine pallidum (Fig. 1a), referred to as the ventral pallidum^44^. This region has a fairly large rostrocaudal extent, can be identified by use of encephalin- and substance P- immunostaining, and distinct subregions can be appreciated based on cytoarchitectonic and hodological criteria (for a review, see Root and colleagues^45^). In particular, the caudal portion of the ventral pallidum extends rostroventrally from the globus pallidus underneath the anterior commissure. Similar to the globus pallidus, this part of the ventral pallidum is also strongly interconnected with the subthalamic nucleus^46^. Rostrally, “finger-like” protrusions of the ventral pallidum extend ventrally into the basal forebrain, interspersed between the olfactory tubercle ventrally and the nucleus accumbens dorsally^44,47^ (Fig. 1a). It should be noted that the ventral pallidum historically was grouped with other basal forebrain regions in an area called “substantia innominata”, whose function was at the time poorly understood (for a historical overview, see Heimer and colleagues^7^). Over time, it has become clear that the dorsal and ventral striatum and pallidum represent two parallel systems, with the dorsal and ventral regions influencing sensorimotor and limbic processes, respectively. Thus, Heimer and colleagues distinguished within the region of the substantia innominata at least three clearly definable nuclei. Most relevant for the present account is the rostral part of the substantia innominata (referred to as the subcommissural substantia innominata), which contains the ventral extension of the globus pallidus, i.e., the ventral pallidum. The caudal parts of the substantia innominata (referred to as the subpallidal or sublenticular substantia innominata) have been identified as part of the so-called ‘extended amygdala’. Finally, in a ventromedial position within the substantia innominata resides a group of large, cholinergic cells sometimes referred to as the basal nucleus or basal nucleus of Meynert^48,49^. Despite these advances, the term “substantia innominata” is still in use in many atlases. In some atlases, this term represents what remains after delineating the ventral pallidum^19^, while in others this area contains the ventral pallidum^13,14^.

The subthalamic nucleus is a lens-shaped region situated dorsomedial to the internal capsule (Fig. 1a,d). It is heavily interconnected with the pallidum^46,50,51^ and represents a relay station in the indirect pathway of the basal ganglia^6^, but is also an important input region for the basal ganglia, receiving extensive input from the thalamus as well as from the cerebral cortex (i.e., hyperdirect pathway)^50,52^. From a functional perspective, the subthalamic nucleus can be subdivided into a dorsolateral (motor), ventromedial (associative) and medial (limbic) part^53^, although in brain atlases it is rarely ever subdivided.

The substantia nigra and ventral tegmental area (VTA; Fig. 1a,e,f) were first described in detail by Dahlström and Fuxe^54^ as part of the rat midbrain dopaminergic system. They identified 12 dopaminergic groups, and the dopaminergic parts of the substantia nigra and ventral tegmental area are numbered, respectively, as the A9 and A10 groups in this scheme. The dopaminergic and non-dopaminergic parts of the substantia nigra are generally referred to as the compact and reticular parts, respectively. This is a simplification often presented in brain atlases, as the compact part has subtypes of dopamine neurons with different connections and patterns of distribution^55–57^. The complex organisation of dopaminergic cell groups in the substantia nigra has led some to propose finer subdivisions such as the dorsal, ventral and medial tier (Fig. 1e)^5,17,56,58^. The reticular part shares several characteristics with the pallidum, together with which it is considered the main output station of the basal ganglia. The dorsolateral pole of the substantia nigra is often referred to as the lateral part^59^. While the division of the substantia nigra into a compact and reticular part are widely accepted, the lateral part is less often recognised in the literature and in atlases, and there are some inconsistencies in the terms and criteria adopted for substantia nigra subregions. For example, the term “substantia nigra” is often used in the literature to refer to the compact part alone^60^, and the compact part is often also referred to as the “dopaminergic substantia nigra”, implying that any dopaminergic cell seen in this region should be considered to belong to the substantia nigra compact part. It is well-known that there is a group of dopaminergic neurons within the region typically called the reticular part, and some thus consider this a ventrally displaced cell group of the pars compacta^58^. The ventral tegmental area is a collective term including several dopaminergic groups at the base of the mesencephalon^58^ that project heavily to ventral parts of the striatum and are involved in motivation and reward processing^61,62^. The VTA is a medial continuation of the substantia nigra pars compacta, but the exact borders between these cell groups have been subject to debate^63^. The exact terminology and subdivisions of the VTA have also been subject to alternative interpretations and confusion over time^57,58,64^. Cells in the substantia nigra pars compacta and ventral tegmental area project to the dorsolateral (mainly caudate-putamen) and ventromedial (mainly nucleus accumbens) parts of the striatum, respectively^65,66^.

#### WHS rat brain atlas v4 basal ganglia regions

**Fig. 2:**
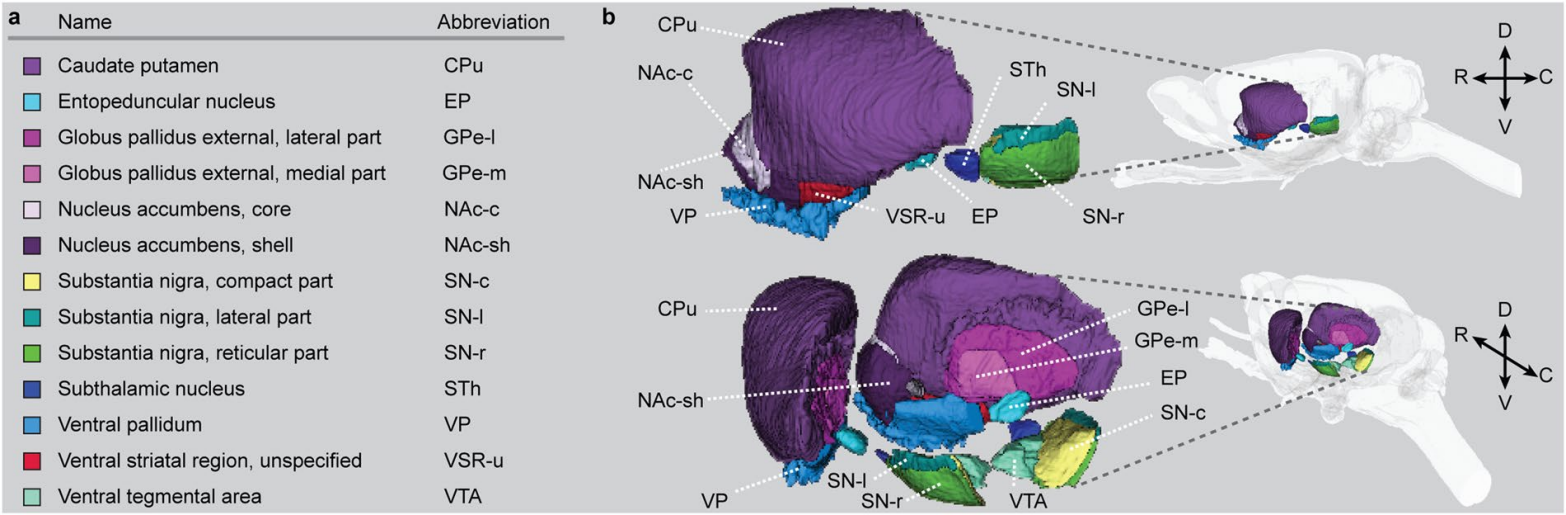
Overview of the basal ganglia regions in the *WHS rat brain atlas v4*. (**a**) List of basal ganglia region colours, terms, and abbreviations in alphabetical order. (**b**) Three-dimensional representation of the basal ganglia regions from a lateral and lateral-caudal view. **Abbreviations:** C, caudal; D, dorsal; R, rostral; V, ventral.

The *WHS rat brain atlas v4*^9^ includes 13 bilateral basal ganglia regions (Fig. 2). An overview of changes across atlas versions are available at the atlas home page (www.nitrc.org/plugins/mwiki/index.php?title=whs-sd-atlas:Annotations). Many of the basal ganglia regions have boundaries that are transitional of nature. As the *WHS rat brain atlas v4* only represents discrete borders, this uncertainty is not captured here. The basal ganglia regions are organised in a hierarchical manner based on their anatomical location. In the following, we describe the boundaries of each region in *WHS rat brain atlas v4*. We focus on the visual appearance of the structures in the different reference data, while the underlying tissue features represented by these are described in the Methods section.

### Striatum

**Fig. 3:**
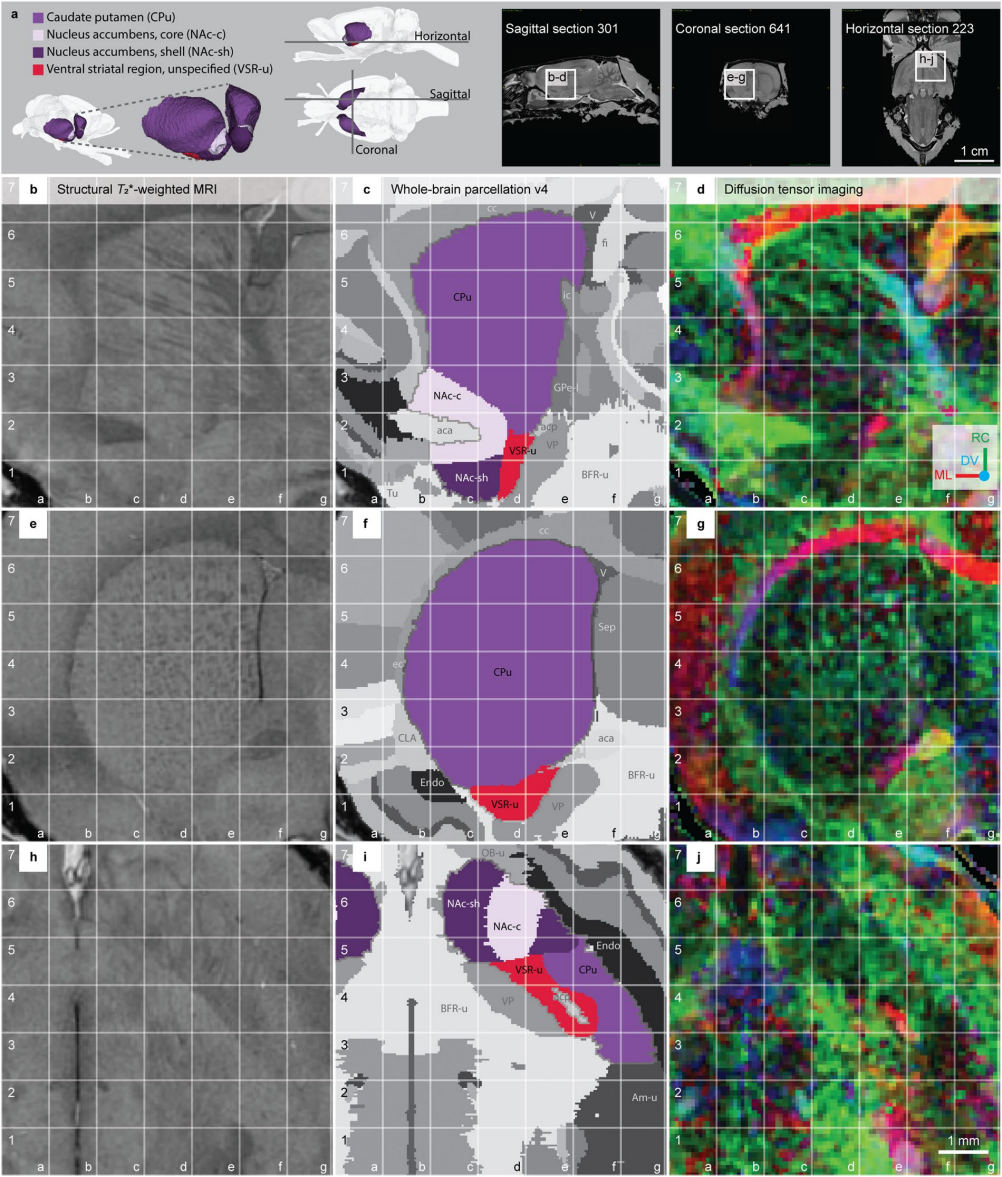
The striatum regions in the *WHS rat brain atlas v4*. (**a**) On the left side: a 3D rendering of the striatum regions in the *WHS rat brain atlas v4*: the caudate-putamen; nucleus accumbens, core; nucleus accumbens, shell; and ventral striatal region, unspecified. In the middle: lateral and horizontal views of 3D renderings with indicators of the horizontal, sagittal and coronal sections shown on the right side. The sagittal, coronal and horizontal sMRI section insets indicate the location and orientation of the MRI slices shown in (**b**–**d,e**–**g,h**–**j**), respectively. (**b,e,h**) Structural *T*_2_*-weighted MRI. (**c,f,i**) Whole-brain parcellation images with the striatum regions in colour and other regions in grey. (**d,g,j**) Diffusion tensor imaging, with DTI orientation colour code inset in (**d**) **Abbreviations:** aca, anterior commisure, anterior limb; acp, anterior commisure, posterior limb; Am-u, amygdaloid area, unspecified; BFR-u, basal forebrain, unspecified; cc, corpus callosum; CLA, claustrum; DV, dorsoventral; ec, external capsule; Endo, endopiriform nucleus; fi, fimbria of the hippocampus; GPe-l, globus pallidus external, lateral part; ic, internal capsule; ML, mediolateral; OB-u, olfactory bulb, unspecified; RC, rostrocaudal; Sep, septal region; Tu, olfactory tubercle; V, ventricle; VP, ventral pallidum.

The striatum is named and recognised by its distinct striated appearance caused by the grouping of myelinated corticofugal fibres descending through the cell-rich grey matter. The striatum comprises the caudate-putamen (CPu), nucleus accumbens core (Nac-c), nucleus accumbens shell (Nac-sh) and ventral striatal region, unspecified (VSR-u) (Fig. 3a). The extension ‘unspecified’ is used in the *WHS rat brain atlas v4* for collective areas that could have been, but were not, further subdivided (see our previous publication^9^ for a discussion of this practice).

**Fig. 4:**
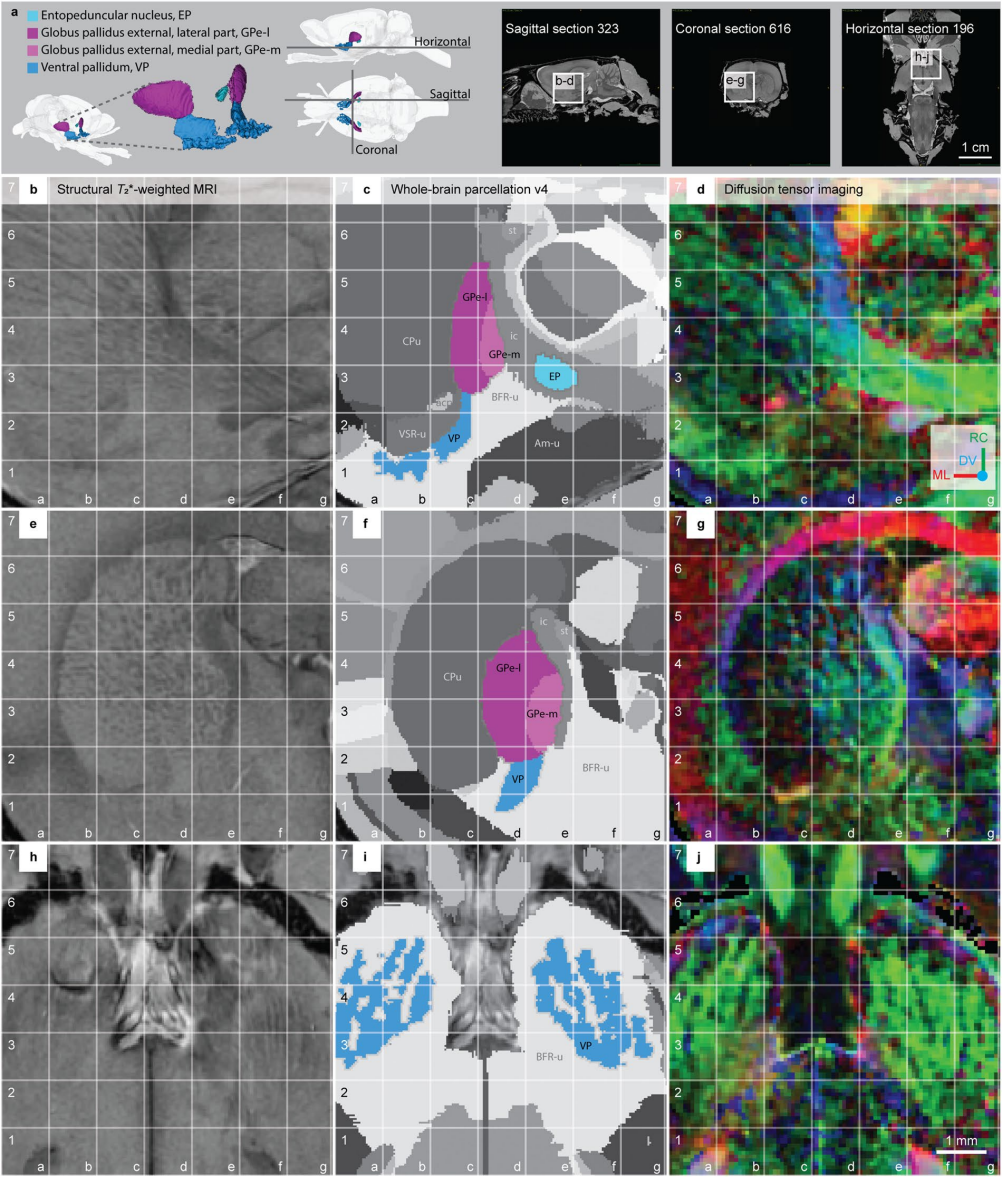
The pallidum regions in the *WHS rat brain atlas v4*. (**a**) On the left side: a 3D rendering of the pallidum regions in the *WHS rat brain atlas v4*: the entopeduncular nucleus; globus pallidus external, lateral part; globus pallidus external, medial part; and ventral pallidum. In the middle: lateral and horizontal views of 3D renderings with indicators of the the horizontal, sagittal and coronal sections shown on the right side. The sagittal, coronal and horizontal sMRI section insets indicate the location and orientation of the MRI slices shown in (**b–d,e–g,h–j**), respectively. (**b,e,h**) Structural *T*_2_*-weighted MRI. (**c,f,i**) Whole-brain parcellation images with the striatum regions in colour and other regions in grey. (**d,g,j**) Diffusion tensor imaging, with DTI orientation colour code inset in (**d**) **Abbreviations:** acp, anterior commisure, posterior limb; Am-u, amygdaloid area, unspecified; BFR-u, basal forebrain, unspecified; CPu, caudate putamen; DV, dorsoventral; ic, internal capsule; ML, mediolateral; RC, rostrocaudal; st, stria terminalis; VSR-u, ventral striatal region, unspecified.

The **caudate-putamen (CPu)** has a curved ellipsoid 3D shape, convex towards lateral and concave towards medial, and is more voluminous rostrally and gradually slanting off towards its caudal end (Fig. 3a). In sMRI, the CPu appears as a relatively bright grey area, perforated by darker bundles of white matter (Fig. 3b,e,h). Rostrally, laterally, dorsally and dorsocaudally the CPu is bordered by white matter fibre tracts, including the corpus callosum, associated subcortical white matter tracts and external capsule (in *WHS rat brain atlas v4* referred to as cc-ec-cing-dwm), which all appear dark grey in sMRI maps, and as brightly coloured areas in DTI maps (Fig. 3d,g,j; in Fig. 4g grid b4-b6, c6, c7-f7, f6-g6). For instance, the corpus callosum appears as bright red voxels in DTI, reflecting the mediolateral orientations of the callosal fibres (Fig. 3d, grid c7-f7 and Fig. 4g, grid c7-f7, f6-g6). Rostromedially and caudally, the CPu abuts the lateral ventricles which in sMRI appear as elongated, narrow dark bands (Fig. 3e, grid f4-f6). The ventral border of the CPu is less clearly distinguishable. Rostroventrally, the CPu share a border with the nucleus accumbens, ventral striatal region, unspecified, endopiriform nucleus and claustrum. The ventral striatal region and nucleus accumbens were challenging to distinguish based on sMRI and DTI: their borders were defined with reference to the literature and other brain atlases (see description in the sections relating to these areas). The endopiriform nucleus and claustrum appear as darker grey areas in sMRI compared to the CPu. The caudomedial part of the CPu shares a border with the globus pallidus external, lateral part (GPe-l) and the internal capsule (included in “corticofugal tract and corona radiata” in the *WHS rat brain atlas*). The GPe-l has a similar appearance to the CPu in sMRI and is thus challenging to distinguish but can be identified using DTI (see description in the section relating to GPe-l). The caudoventral border of the CPu towards the amygdalar nuclei (ventrolaterally: basolateral nucleus, ventromedially: central nucleus, both delineated as amygdaloid area, unspecified in the *WHS rat brain atlas*) can be discerned in the coronal and sagittal planes by the more striated appearance of the CPu. The caudal border of the CPu abutting the hippocampal region is defined by the ventricular system and white matter (Fig. 3b, grid f5-f7).

The nucleus accumbens (NAc) consist of two parts, the **nucleus accumbens core (NAc-c)** and the **nucleus accumbens shell (NAc-sh)**. The rostral tip of the nucleus accumbens, by some referred to as the “rostral pole”, is included in the shell. Both the NAc-c and NAc-sh are present across the rostro-caudal extent of the NAc and appear in sMRI as areas with medium grey voxels, generally brighter than surrounding regions (Fig. 3b, grid c3,d2). In DTI, the NAc appears darker than the surrounding regions (Fig. 3d, grid c1-c2, d1-d2). Laterally, the NAc abuts on the endopiriform nucleus, which appears more intensely green (Fig. 3j, grid d6 versus e6–7). Ventrally, the ‘olfactory tubercle (Tu)’ (included in “basal forebrain region, unspecified” in *WHS rat brain atlas*) appears slightly darker-grey in sMRI and DWI (Fig. 3b, grid b1-c1). The NAc, specifically the NAc-c, is perforated by a darker-grey bundle of white matter (the anterior commissure, anterior limb; Fig. 3b–d, grid b2-c2). The dorsal borders of the NAc are difficult to identify due to similarity with the caudate-putamen. The dorsal and dorsolateral border of the NAc towards the CPu was mainly extrapolated by visual comparison to literature and other atlases. However, the lateral aspect of this border can be identified as a thin dark rim in sMRI, which to some extent also is detectable in DWI. The border between the NAc-c and NAc-sh is difficult to define, but the slightly brighter appearance of NAc-c compared to the NAc-sh in DWI helps define the ventromedial part of their border. Thus, the separation of the NAc-c and NAc-sh was mainly achieved using DWI, aided by literature and comparison to histological material and other atlases.

The **ventral striatal region, unspecified (VSR-u)** is a collective area with striatal appearance, situated ventral to the CPu and caudal to the NAc. In sMRI, the VSR-u can be distinguished based on its relatively darker and less striated appearance than the CPu (Fig. 3e, grid d1-d2 and e1-e2; Fig. 3h, grid d5-e5, e4-f4). Its caudal end is approximately at the level where the septum separates from the basal forebrain region ventrally. In DTI, the VSR-u appears darker and displays a mixture of low intensity, multi-coloured voxels, compared to the neighbouring ventral pallidum and the collective region basal forebrain, unspecified. The border of the VSR-u towards the white matter (anterior commissure, anterior limb; aca) is readily seen as the latter appears dark grey in sMRI and with red voxels in DTI (Fig. 3h–j, grid e4).

### Pallidum

**Fig. 5:**
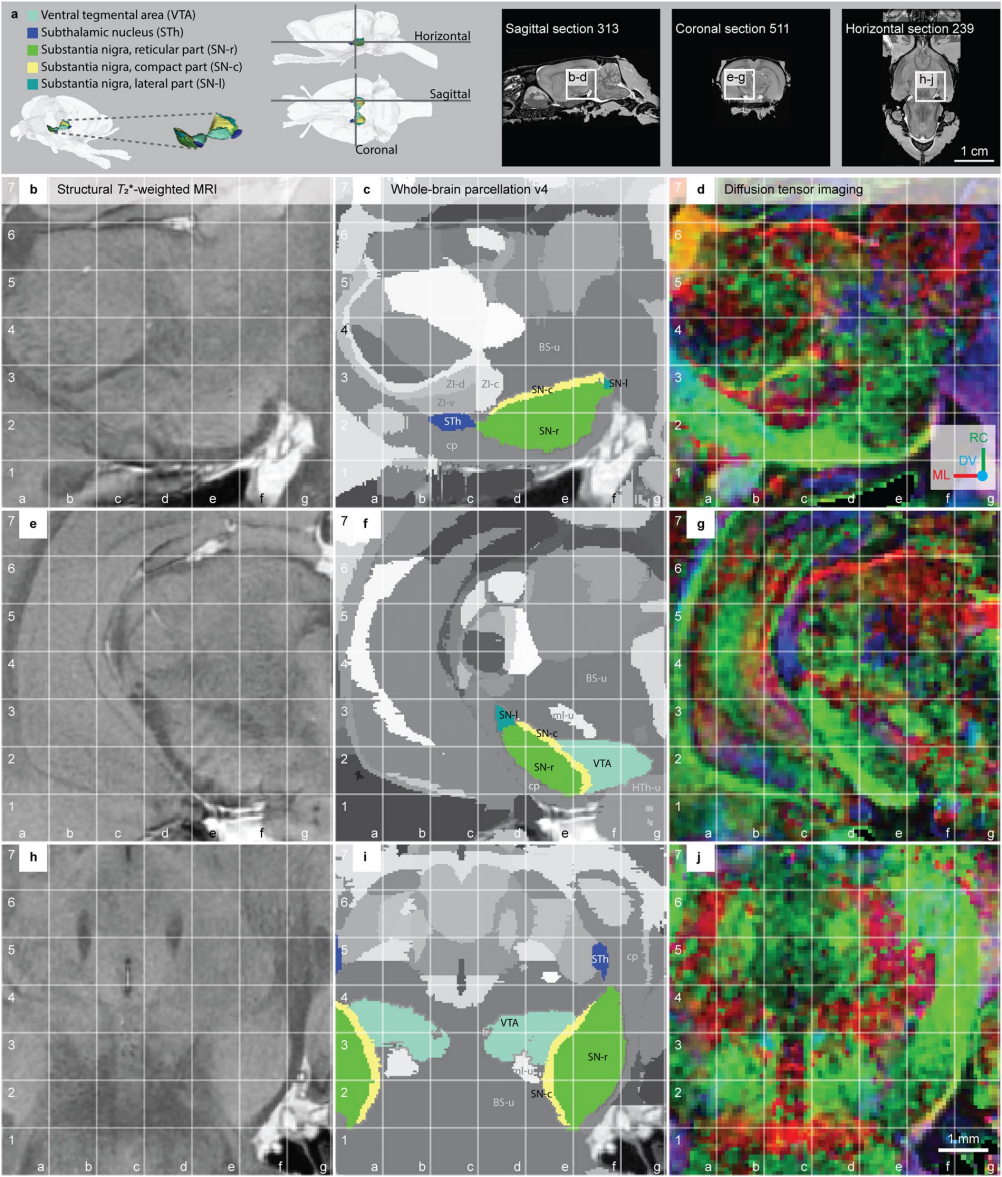
The subthalamic nucleus, substantia nigra and ventral tegmental area in the *WHS rat brain atlas v4*. (**a**) On the left side: a 3D rendering of the ventral tegmental area, subthalamic nucleus, substantia nigra reticular, compact and lateral parts in the *WHS rat brain atlas v4*. In the middle: lateral and horizontal views of 3D renderings with indicators of the horizontal, sagittal and coronal sections shown on the right side. The sagittal, coronal and horizontal sMRI section insets indicate the location and orientation of the MRI slices shown in (**b**–**d,e**–**g,h**–**j**), respectively. (**b,e,h**) Structural *T*_2_*-weighted MRI. (**c,f,i**) Whole-brain parcellation images with the striatum regions in colour and other regions in grey. (**d,g,j**) Diffusion tensor imaging, with DTI orientation colour code inset in (**d**) **Abbreviations:** BS-u, brainstem, unspecified; cp, cerebral peduncle; DV, dorsoventral; HTh-u, hypothalamic region, unspecified; ML, mediolateral; ml-u, medial lemniscus, unspecified; RC, rostrocaudal; ZI-c, zona incerta, caudal part; ZI-d, zona incerta, dorsal part; ZI-v, zona incerta, ventral part.

The pallidum consists of the globus pallidus external, lateral part (GPe-l), the globus pallidus external, medial part (GPe-m), the ventral pallidum (VP) and the entopeduncular nucleus (EP) (Fig. 4a). The globus pallidus external (GPe) consist of two parts, the **globus pallidus external, medial part (GPe-m)** and **globus pallidus external, lateral part (GPe-l)**. We chose to include the term “external” to make it clear that both these regions are part of the segment often referred to as the external globus pallidus in other atlases. We avoid the term “internal”, which has been used variably in the past and could contribute to confusion. The medial border of the GPe with the internal capsule is distinguished by the white matter, appearing darker in sMRI and brighter (blue and green) in FA (Fig. 4e–g, grid e3-e4). The dorsolateral border towards the CPu is more difficult to distinguish in sMRI (see, above) due to the striated appearance of the tissue in both regions. In DTI maps, the GPe-l features intense blue and green coloured voxels, while the CPu appears with widely dispersed green voxels among low-intensity red and blue voxels (Fig. 4g, compare grid c4, c5, d5 to d3, e4). Rostrally, the posterior limb of the anterior commissure and the associated interstitial nucleus defines the ventral border of the GPe, which appears slightly brighter in sMRI. More caudally, the ventral border of the GPe towards the VP and extended amygdala can be identified based on the striated appearance of the GPe, with high densities of elongated, dorsoventrally oriented fibre bundles, contrasting the more homogeneous appearance of the surrounding regions (Fig. 4b, grid c3 and Fig. 5e, grid d2-e2). The caudal border of the GPe towards the stria terminalis and internal capsule is visible from the distinctly contrasting white matter signature, with dark sMRI voxels and intensely coloured DTI voxels (Fig. 4b–d, grid e4, e5). The subdivision of the GPe into a GPe-l and GPe-m can be seen in the caudal third of the GPe, where the fibre bundles in the GPe-l appear homogeneously distributed, giving a slightly darker appearance in sMRI compared to the GPe-m (Fig. 4e, grid d3-d4, e3-e4). From DTI maps, subtle differences in the organisation of the fibre bundles perforating the GPe can be observed. In the GPe-l, fibre bundles are more widely dispersed (Fig. 4d, grid c4-c5, grid d3-d4), while in GPe-m, fibres converging into the obliquely oriented corticofugal tract, visible as confluent blue-green voxels merging to form a rostrocaudally oriented fibre tract (intensely green; Fig. 4d, grid d4 and Fig. 5g, grid e3-e4).

The **entopeduncular nucleus (EP)** is a small region situated in the white matter of the internal capsule. In sMRI, the EP is visible as an area with bright voxels perforated by strings of dark voxels (fibre bundles) at the medioventral edge of the internal capsule (Fig. 4b–d, grid e3-e4). The rostroventral border of the EP with other grey matter regions was challenging to distinguish and was thus made to follow the border of the internal capsule. More caudally, the EP is gradually embedded in the white matter. The EP is also visible in the DTI maps as less intensively coloured voxels dispersed among the bright green voxels of the fibre bundles in the internal capsule (Fig. 4d, grid e3-e4).

The **ventral pallidum (VP)** is a ventral extension of the GPe (Fig. 4a). Dorsally it is bordered by the NAc-sh, basal forebrain region, anterior commissure, and VSR-u. At its rostral extreme, the VP can be recognised embedded in the so-called “striatal bridges” that extend ventrally from the nucleus accumbens shell towards the brain surface, and visible as finger-like protrusions appearing as intensely green coloured voxels reflecting the rostrocaudal orientation of fibres, in contrast with the dispersed multi-coloured voxels of the surrounding area (Fig. 4j). Thus, the VP extends ventrally and rostrally, intermingling with the striatal extensions from the NAc-sh into the olfactory tubercle. More caudally, the medial part of the VP gradually increases in size and the striatal bridges end. In this part, the VP appears more homogeneous in DTI and is not easily distinguished from surrounding regions (Fig. 4d, grid c2 compared with a1-a2 and b1-b2). However, in sMRI, the caudal part of the VP appears brighter than its surrounding regions: the anterior commissure dorsally, the ventral part of the striatum dorsolaterally, and the basal forebrain medially, ventrally, and caudally (Fig. 4b).

### Subthalamic nucleus

The **subthalamic nucleus (STh)** is a small region situated dorsally and medially to the cerebral peduncle, intercalated between the cerebral peduncle and the zona incerta (Fig. 5b–d, grid c2). The cerebral peduncle in the *WHS rat brain atlas*, part of the “descending corticofugal pathways”, surrounds the STh rostrally, ventrally, and laterally. In DTI maps, the STh is visible as a group of red voxels with lower colour intensity than the surrounding white matter featuring high intensity green voxels (Fig. 5h–j, grid f5). Rostromedially, the STh has a border with the ‘zona incerta, ventral part’, and dorsoventrally it borders the ‘basal forebrain, unspecified’. The STh abuts the SN-r caudally (Fig. 5b–d, grid c2-d2) while the ‘brainstem’ makes up the rest of its medial, dorsal, and caudal borders.

### Substantia nigra

The substantia nigra (SN) consists of three parts, the **substantia nigra compact part (SN-c),** **substantia nigra reticular part (SN-r)**, and **substantia nigra lateral part (SN-l)**. In sMRI, the SN-r and SN-c border is distinguished from the latter appearing darker (Fig. 5b,e, grid e3). The SN-l is situated dorsolaterally in the SN, appearing slightly darker in sMRI compared to the SN-r (Fig. 5e, grid d3). The SN has a border with the STh rostrally and the VTA rostromedially, while the ventral, lateral and dorsolateral border is given by the adjacent white matter of the descending cerebral peduncle. These borders are particularly visible in DTI, where the SN features a mixture of colours with lower intensity, contrasting the more homogenous bright green appearance of the cerebral peduncle (Fig. 5g, grid d2-d3, e1-e2 and f1). The medial, dorsal, and caudal aspects of the SN border various brainstem regions (‘brainstem, unspecified’ in *WHS rat brain atlas*).

### Ventral tegmental area

**Fig. 6:**
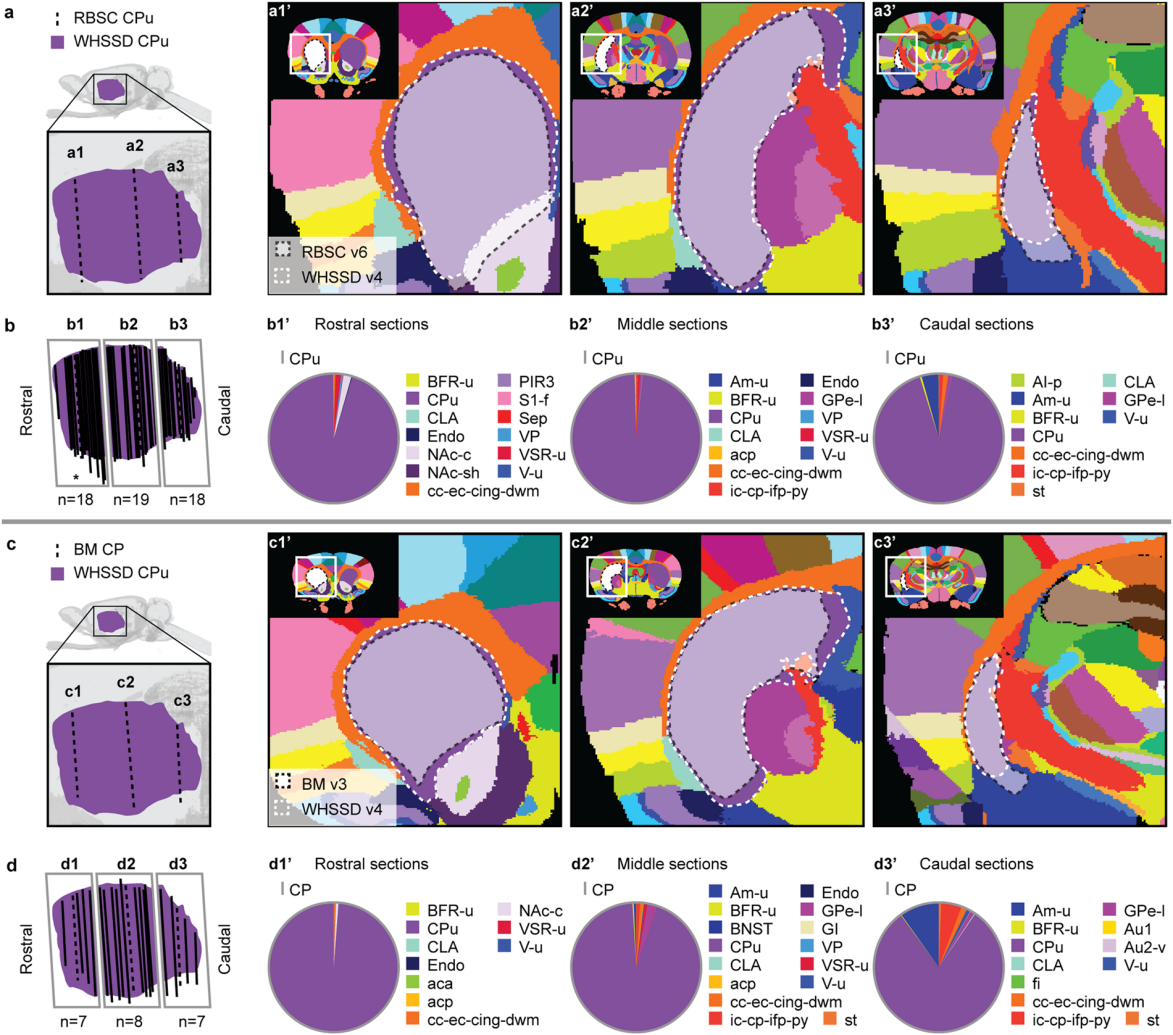
Quantitative spatial analysis of the caudate-putamen (CPu) across rat brain atlases. (**a**) Lateral view of the *WHS rat brain atlas (WHSSD)* CPu (purple), with magnified panel showing the spatial position and angle of three selected atlas plates (dashed lines) from rostral (a1), middle (a2) and caudal (a3) parts of *The Rat Brain in Stereotaxic Coordinates (RBSC)* CPu. The *WHSSD* cut to correspond with the three selected atlas plates from *RBSC* (a1’-a3’), showing the spatial correspondence of the CPu (black dashed line filled with a transparent white colour) with regions in the *WHSSD*. The *WHSSD* CPu is highlighted for comparison (white dashed lines). (**b**) Lateral view of the *WHSSD* CPu, showing the spatial position and angle of the selected atlas plates (a1-a3, dashed lines) and the remaining *RBSC* atlas plates including CPu annotations (solid lines). The boxes (b1-b3) indicate the three rostro-caudal parts used for analysis of the quantitative spatial overlap of the *RBSC* CPu with regions in the *WHSSD*. The pie charts (b1’-b3’) show the proportion of the *RBSC* CPu annotation situated in regions of the *WHSSD*. (**c,d**) Selected atlas plates (c1-c3, c1’-c3’), spatial position (d1-d3) and pie charts (d1’-d3’) for the *Brain Maps (BM)* CP annotation in relation to *WHSSD* regions, as described for (**a,b**). Note that the atlas plates from *BM* are registered with a mediolateral angle of 4 degrees, which in c1-c3 and d1-d3 gives an illusion of a dorsoventral angle in some atlas plates. **Abbreviations:** aca, anterior commissure, anterior limb; acp, anterior commissure, posterior limb; AI-p, agranular insular cortex, posterior area; Am-u, amygdaloid area, unspecified; Au1, primary auditory cortex; Au2-v, secondary auditory area, ventral part; BFR-u, basal forebrain region, unspecified; BM, *Brain Maps*; BNST, bed nucleus of the stria terminalis; cc-ec-cing-dwm, corpus callosum and associated subcortical white matter; CPu, caudate putamen; CLA, claustrum; Endo, endopiriform nucleus; fi, fimbria of the hippocampus; GI, granular insular cortex; GPe-l, globus pallidus external, lateral part; ic-cp-ifp-py, corticofugal tract and corona radiata; NAc-c, nucleus accumbens, core; NAc-sh, nucleus accumbens, shell; PIR3, piriform cortex, layer 3; RBSC, *The Rat Brain in Stereotaxic Coordinates*; S1-f, primary somatosensory area, face representation; Sep, septal region; st, stria terminalis; VP, ventral pallidum; V-u, ventricular system, unspecified; VSR-u, ventral striatal region, unspecified; WHSSD, *WHS rat brain atlas*.

The **ventral tegmental area (VTA)** includes several smaller neuronal groups. In sMRI, the VTA appears as a relatively homogeneous region with a medium signal intensity (Fig. 5e, grid f2-g2). The individual subnuclei are difficult to differentiate, therefore the VTA was delineated as a single region. The VTA is situated medially to the rostral half of the SN. The lateral border of the VTA with the SN is readily identified from the slightly brighter appearance of SN in sMRI (Fig. 5h, grid d3-d4 and e3-e4). In DTI, the VTA is dominated by red voxels, indicating mediolateral diffusion orientations, distinguishing it from the green and purple appearance of the SN and brainstem regions (Fig. 5g, grid e3; Fig. 6j, grid d4, e4). The medial, dorsal, ventral, rostral and caudal borders of the VTA is with the brainstem and can be distinguished by the relatively brighter appearance of the VTA in sMRI (Fig. 5h, grid d4, e4) and DWI. The caudal border of the VTA is clearly distinguished from the higher FA values in the neighbouring brainstem and white matter tracts (Fig. 5h-j, grid d3-d4, e3-e4).

### Overview of basal ganglia annotations and terms across atlas versions

We assessed how basal ganglia regions are named and annotated across all versions of *The Rat Brain in Stereotaxic Coordinates* and *Brain Maps*, summarised in Tables 1, 2. We found that annotations and naming conventions not only differ between atlases, but also change across atlas versions, in most cases due to inclusion of new subregions, but occasionally also as straightforward changes of nomenclature. Most of the changes noted here are not described in the atlas introductions and are only apparent when comparing atlas plates at similar levels. Detailed descriptions of all the changes are included in our database of atlas regions^67^.

**Table 1:** Basal ganglia regions in *The Rat Brain in Stereotaxic Coordinates* versions 1–7.

Basal Ganglia annotations in *The Rat Brain in Stereotaxic Coordinates*									
Hier.	Abb.	Term (version 6)	Version						
			1	2	3	4	5	6	7
STR	Acb	accumbens nucleus	n	ma	r				
	AcbC	accumbens nucleus, core		n	mi	u	mi	u	u*
	AcbSh	accumbens nucleus, shell		n	mi	u	mi	mi	mi*
	AcbR	accumbens nucleus, rostral pole					n	r	
	FStr	fundus striati	n	ma	r				
	CPu	caudate putamen (striatum)	n	mi	mi	u	mi	u	mi
	IPAC	interstitial nucleus of the posterior limb of the anterior commissure			n	u	ma	u	u
	IPACL	interstitial nucleus of the posterior limb of the anterior commissure, lateral part			n	u	ma	u	u
	IPACM	interstitial nucleus of the posterior limb of the anterior commissure, medial part			n	u	ma	u	u
	LAcbSh	lateral accumbens shell			n	u	mi	u	mi*
	LSS	lateral stripe of the striatum			n	u	ma	u	u
PAL	EGP	external part of globus pallidus							n
	IGP	internal part of globus pallidus							n
	GP	globus pallidus	n	mi	u*	u*	mi*	mi	r
	EP	entopeduncular nucleus	n	ma	ma*	u*	ma*	u	u
	SI	substantia innominata	n	ma	ma	u	r		
	SIB	substantia innominata, basal part			n	u	mi	u	mi
	SID	substantia innominata, dorsal part			n	u	r		
	SIV	substantia innominata, ventral part			n	u	r		
	VP	ventral pallidum	n	ma	ma	u	mi	u	u
SN	SNC	substantia nigra, compact part	n	ma	r				
	SNCD	substantia nigra, compact part, dorsal tier			n	u	ma	u	u
	SNCM	substantia nigra, compact part, medial tier			n*	u*	ma	u	u
	SNCV	substantia nigra, compact part, ventral tier			n	u	ma	u	u
	SNL	substantia nigra, lateral part	n	ma	u	u	mi	u	u
	SNR	substantia nigra, reticular part	n	mi	mi	u	mi	u	u
	SNRDM	substantia nigra, reticular part, dorsomedial tier			n	u	r		
	SNRVL	substantia nigra, reticular part, ventrolateral tier			n	u	r		
VTA	PBP	parabrachial pigmented nucleus of the VTA	n*	u*	ma*	u*	ma	mi	u*
	PIF	parainterfascicular nucleus of the VTA					n	u	u*
	PN	paranigral nucleus of the VTA	n*	u*	u*	u*	ma	u	u*
	VTA	ventral tegmental area	n	mi	mi	u	ma	u	u
	VTAR	ventral tegmental area, rostral part		n	r		n	u	u
STN	STh	subthalamic nucleus	n	mi	mi	u	mi	u	u

Changes in annotations across version are coded using the following abbreviations: n, new; u, unchanged; mi, minor changes; ma, major changes; r, replaced. The abbreviations and terms in the left column are from version 6. * The region has another term in other versions. AcbC v7, accumbens nucleus, core region; AcbSh v7, accumbens nucleus, shell region; GP v3–4 lateral globus pallidus (LGP); GP v5, external globus pallidus (EGP); EP v3–4, medial globus pallidus (MGP); EP v5, internal globus pallidus (intrapeduncular nucleus) (IGP); LAcbSh v7, lateral accumbens, shell region; PBP v1–4, parabrachial pigmented nucleus; PBP v7, parabrachial pigmented nucleus of the ventral tegmental area; PIF v7, parainterfascicular nucleus of the ventral tegmental area; PN v1–4, paranigral nucleus; PN v7, paranigral nucleus of the ventral tegmental area; SNCM v3–4, substantia nigra, medial part (SNM). Note that there is a substantial change in the rat brain reference data between version 4 and 5. The regions are here categorised as belonging to the striatum (STR), pallidum (PAL), substantia nigra (SN), ventral tegmental area (VTA), or subthalamic nucleus (STN). **Abbreviations:** Abb, abbreviation; Hier, hierarchical parent region.

**Table 2:** Basal ganglia regions in *Brain Maps*: Structure of the Rat Brain version 1–4.

Basal ganglia annotations in *Brain Maps: Structure of the Rat Brain*						
Hier.	Abb.	Term (version 3)	Version			
			1	2	3	4
STR	CP	Caudoputamen	n	u	mi	u
	ACB	Nucleus accumbens	n	u	u	u*
	FS	Striatal fundus	n*	mi*	u	u
PAL	GPe	Globus pallidus external segment	n*	u*	u	u*
	GPi	Globus pallidus internal segment	n*	u*	u	u*
	SI	Substantia innominata	n	mi	mi	u*
SN	SNc	Substantia nigra, compact part	n	u	u	u
	SNr	Substantia nigra, reticular part	n	u	u	u
VTA	VTA	Ventral tegmental area	n	u	u	u
STN	STN	Subthalamic nucleus	n	u	u	u

Changes in annotations across version are coded using the following abbreviations: n, new; u, unchanged; mi, minor changes; ma, major changes; r, replaced. The abbreviations and terms in the left column are from version 3. * The region has another term in other versions. ACB v4, Accumbens nucleus; FS v1–2, Fundus of the striatum; GPe v1–2 and 4, Globus pallidus lateral segment (GPl); GPi v1–2 and 4, Globus pallidus medial segment (GPm); SI v4, Innominate substance. The regions are here categorised as belonging to the striatum (STR), pallidum (PAL), substantia nigra (SN), ventral tegmental area (VTA), or subthalamic nucleus (STN). **Abbreviations:** Abb, abbreviation; Hier, hierarchical parent region.

### The rat brain in stereotaxic coordinates (Paxinos & Watson)

Across the seven published versions of *The Rat Brain in Stereotaxic Coordinates*, we identified 33 regions that are here considered as part of or closely related to the basal ganglia (Table 1). In the following text, these regions are categorised as belonging to the striatum, pallidum, subthalamic nucleus, substantia nigra, or ventral tegmental area.

The striatal region includes the caudate putamen (striatum) (CPu), accumbens nucleus (Acb), fundus striati (FS), lateral accumbens shell (LAcbSh), interstitial nucleus of the posterior part of the anterior commissure (IPAC) and lateral stripe of striatum (LSS). The caudate putamen (striatum) (CPu) appears in all versions. The term is identical across all versions, while there are minor changes to the annotations in version 2, 3, 5, and 7; these changes involve small adjustments of the ventral border against ventral striatal regions rostrally and/or the amygdala caudally. The accumbens nucleus (Acb) is annotated as a single structure in version 1, but is subdivided into the accumbens nucleus, core (AcbC) and accumbens nucleus, shell (AcbSh) in all subsequent versions. The accumbens nucleus, rostral pole (AcbR) is only delineated in version 5; in other versions, this rostral portion is considered part of the shell region. The AcbC and AcbSh are fairly consistently annotated across versions, with minor changes in versions 3 and 5 for the AcbC and in versions 3, 5, 6 and 7 for the AcbSh. Most notably, the shell region seems to have a larger caudal extent in version 5 and following versions, where it extends dorsally and medially into areas previously considered bed nucleus of the stria terminalis.

In addition to the nucleus accumbens core and shell, the atlas includes a ventral striatal area called the fundus striati (FS) in versions 1 and 2, which is partly replaced by the lateral accumbens shell (LAcbSh) in versions v3–7. Curiously, the LAcbSh is introduced as an area corresponding “to a region often called fundus striati”^16^, but only the rostral part of the FS is replaced by the LAcbSh. The caudal part of the FS is from version 3 onwards replaced by the interstitial nucleus of the posterior limb of the anterior commissure (IPAC). While the IPAC may not be considered part of the basal ganglia, at least part of it is reminiscent of the striatum in terms of cytoarchitecture^68^, and we include it in our descriptions due to its proximity to the CPu and Acb. The IPAC is, as the term implies, situated around the posterior limb of the anterior commissure. Caudally, the IPAC is separated into a lateral (IPACL) and medial (IPACM) segment, while rostrally the region is simply labelled IPAC. We here refer to these regions collectively as the IPAC complex. The delineation of the IPAC complex changes substantially between versions 4 and 5. Specifically, it is more extensive in early versions (v3–4), with a more prominent part dorsal to the commissural limb and extending more caudally than in later versions (v5–7). In these later versions, most of the area previously occupied by the IPACL dorsal to the commissural limb is defined as CPu, while the most caudal part of the IPACL from earlier versions is named amygdalostriatal transition area. Lastly, the later versions include the lateral stripe of striatum (LSS; v3–7), a very small region embedded in the transition between the CPu and Acb.

The pallidal region includes the globus pallidus (GP), entopeduncular nucleus (EP), ventral pallidum (VP) and substantia innominata (SI). The external part of the globus pallidus is referred to as external part globus pallidus (EGP; v5, 7), lateral globus pallidus (LGP; v3–4), or globus pallidus (GP; v1, 6). The entopeduncular nucleus (EP) is referred to as the internal globus pallidus (intrapeduncular nucleus) (IGP; v5), medial globus pallidus (MGP; v3–4), or entopeduncular nucleus (EP; v1, 6–7). For simplicity, we refer to these regions by their terms from version 6 (GP and EP) in the following section. The GP is quite consistently delineated across versions; however, in version 7, it is subdivided into a medial and lateral part termed internal and external part of the globus pallidus (IGP and EGP), respectively. This change was made in response to the debates concerning the identity of EP as the murine homologue of the internal globus pallidus (see section on “Overview of basal ganglia regions, nomenclature and hierarchy”). The EP annotation changes in shape and increases in size in both version 2 and 3. There are also some changes in version 5, where it is smaller and less round rostrally and the rostrocaudal extent appears more restricted compared to version 4.

The ventral pallidum (VP; v1–7) is present in all atlas versions. While the term is identical across versions, the annotations have major changes in version 2 and 3, and a minor change in version 5. The substantia innominata (SI) is annotated as a large collective region. In version 3, most of the rostral SI is annotated as the substantia innominata, basal part (SIB), while the caudal SI is subdivided into a dorsal (SID) and ventral (SIV) part. The SID and SIV are in later versions (version 5 onwards) replaced by the sublenticular extended amygdala, while what remained of the SI was relabelled as SIB.

The subthalamic nucleus (STh) is present and consistently named across all atlas versions. Minor changes in its borders are apparent in version 2, 3 and 5.

The substantia nigra compact part (SNC), substantia nigra reticular part (SNR) and substantia nigra lateral part (SNL) are present in all versions, while subregions of the SNC and SNR vary. The SNC was annotated as a single region in versions 1–2. From version 3 onwards, the SNC is subdivided into dorsal, medial, and ventral parts (SNCD, SNCM and SNCV, respectively). Notably, part of the rostroventral SNC from versions 1–2 is replaced by the parabrachial pigmented nucleus (PBP) in later versions. The SNR is rarely subdivided, but dorsomedial (SNRDM) and ventrolateral tier (SNRVL) are indicated in one atlas plate (diagram 38) in versions 3–4. The SNL undergoes major changes between version 1 and 2 and minor changes between version 4 and 5 but is otherwise similarly annotated across versions.

The ventral tegmental area constitutes a group of regions (here referred to as the VTA complex) including the paranigral nucleus (PN), the parainterfascicular nucleus (PIF), the parabrachial pigmented nucleus (PBP) and the ventral tegmental area, rostral part (VTAR), and the ventral tegmental area (VTA; used to annotate the parts that remain after identifying the other subregions). In early versions (v1–4), the VTA complex appears as an amorphous area, with the labels “VTA”, “PN” and PBP” annotating the collective area. In version 5, the subregions of the VTA complex were changed to include borders for all subregions. Most of the general “VTA” term has been replaced by specifically named parts in this version, both PBP and PN show major changes in version 5, in which the PIF is annotated for the first time. The authors of the atlas state in version 6 that the VTA is fully represented by its specifically named parts, solving “the challenge where this region was called VTA and thus giving the impression that this constituted the whole region”^19^. However, there is still an area labelled VTA in the most caudal part of the VTA complex in version 6 and 7. The VTAR is first included in version 2, then replaced in version 3 and re-introduced and consistently present in version 5 onwards.

### Brain Maps: structure of the rat brain (Swanson)

Across the four versions of *Brain Maps: Structure of the Rat Brain* (hereafter referred to as *Brain Maps*), we identified 10 regions that are here considered as part of or closely related to the basal ganglia (Table 2). In the following text, these regions are categorised as belonging to the striatum, pallidum, subthalamic nucleus, substantia nigra, or ventral tegmental area.

The striatal region includes the caudoputamen (CP), nucleus accumbens (ACB), and striatal fundus (FS). Minor changes in the CP annotation can be observed in version 3, where the caudoventral border towards the amygdala and substantia innominata have been revised. The ACB annotation is identical across versions, while the lateral borders of the FS are slightly changed in version 2 due to changes in the adjacent external capsule.

The pallidal regions include the globus pallidus internal segment (GPi) and globus pallidus external segment (GPe). These are in some versions referred to as globus pallidus medial segment (GPm) and globus pallidus lateral segment (GPl), respectively. There is no ventral pallidum annotation as this region is included in a larger collective area referred to as substantia innominata (SI; v1–3) or innominate substance (v4). Minor changes to the lateral and caudal extent of the SI can be seen in version 2 and 3, respectively.

The substantia nigra, compact part (SNc) and substantia nigra, reticular part (SNr) are annotated without further subdivisions, as is the ventral tegmental area (VTA). The subthalamic nucleus (STN), SNc, SNr and VTA are all consistently named and annotated across the atlas versions.

### Comparison of basal ganglia regions across commonly used rat brain atlases

Across commonly used rat brain atlases and their versions, the basal ganglia regions not only differ in subdivisions and terms, they also vary in position and size. To investigate the degree of spatial correspondence of basal ganglia delineations across rat brain atlases, we co-registered atlas plates to the 3D reference dataset of *WHS rat brain atlas*. Below, we describe and visualise how basal ganglia regions in *The Rat Brain in Stereotaxic Coordinates* and *Brain Maps* compare to those defined in *WHS rat brain atlas*. We first summarise the quantitative spatial overlap of basal ganglia regions in *The Rat Brain in Stereotaxic Coordinates* (6^th^ version)^19^ and *Brain Maps* (3^rd^ version)^21^ with the *WHS rat brain atlas* (4^th^ version). To complement our quantitative findings, we qualitatively describe where differences occur, also making note of substantial changes across versions of each atlas. For *Brain Maps* version 3, the spatially registered atlas plates are available through an online viewer via the EBRAINS Knowledge Graph^69^. Summary data on the quantitative overlap for all regions and the complete underlying datasets for both qualitative and quantitative analyses are available on EBRAINS Knowledge Graph^67,70^.

It is important to note that the stereotaxic atlases investigated here do not cover regions entirely, but rely on sampling a representative subset throughout the brain. *The Rat Brain in Stereotaxic Coordinates* (version 6) has a sampling ratio of 29–67% (average ratio: 37%) depending on the region. The sampling ratio for regions in *Brain Maps* (version 3) range from 15–24%, with an average of 19%. The sampling ratio of each basal ganglia region in these two atlas versions, calculated based on their anteroposterior extent and the number of sections they appear in, can be found in Table 3 (for *The Rat Brain in Stereotaxic Coordinates* v6) and Table 4 (for *Brain Maps* v3).

**Table 3:** Sampling frequency of regions in *The Rat Brain in Stereotaxic Coordinates* version 6.

Basal Ganglia annotations in *The Rat Brain in Stereotaxic Coordinates* version 6							
Hier.	Abb.	Rostral end	Caudal end	Total length	# sampled sections	# total sections	Sample ratio
STR	AcbC	3	0,48	2,52	19	63	30%
	AcbSh	3,24	0,48	2,76	20	69	29%
	CPu	2,76	−3,96	6,48	55	162	34%
	IPAC	0,6	−1,44	1,08	9	27	33%
	IPACL	−0,24	−1,2	0,96	9	24	38%
	IPACM	−0,24	−1,2	0,96	9	24	38%
	LAcbSh	2,16	0,84	1,32	12	33	36%
	LSS	2,28	−0,6	2,88	24	72	33%
PAL	GP	−0,24	−3,12	2,88	25	72	35%
	EP	−1,92	−2,76	0,84	8	21	38%
	VP	3	−1,08	4,08	32	102	31%
SN	SNCD	−4,56	−6,6	2,04	18	51	35%
	SNCM	−5,76	−6,6	0,84	8	21	38%
	SNCV	−5,76	−6,6	0,84	8	21	38%
	SNL	−4,56	−6,48	1,92	17	48	35%
	SNR	−4,36	−6,72	2,36	21	59	36%
VTA	PBP	−4,56	−6,48	1,92	17	48	35%
	PIF	−5,52	−6,6	1,08	10	27	37%
	PN	−5,2	−6,48	1,32	12	33	36%
	VTA	−6,72	−6,84	0,12	2	3	67%
	VTAR	−4,68	−5,04	0,36	4	9	44%
STN	STh	−3,12	−4,2	1,08	10	27	37%

The basal ganglia regions in the Rat Brain in Stereotaxic Coordinates (version 6) are listed with their abbreviation under Abb.) and sorted into broad hierarchical groupings as defined in the main paper. The table gives the rostral and caudal end of each structure relative to Bregma, extracted from the atlas plates. The total length was estimated as the distance (in mm) between the rostral and caudal end. The number of sampled sections are given, and corresponds to the number of atlas plates containing the region annotation. The total number of sections was calculated based on the total length and the section thickness (40 µm). Lastly, the sample ratio gives the ratio of sampled sections to estimated total number of sections.

**Table 4:** Sampling frequency of regions in *Brain Maps* version 3.

Basal ganglia annotations in *Brain Maps: Structure of the Rat Brain* version 3							
Hier.	Abb.	Rostral end	Caudal end	Total length	# sampled sections	# total sections	Sample ratio
STR	CP	2,15	−3,7	5,85	22	146	15%
	ACB	2,8	0,45	2,35	7	59	12%
	FS	1,2	−0,83	2,03	10	51	20%
PAL	GPe	−0,26	−2,85	2,59	11	65	17%
	GPi	−1,08	−2,45	1,37	6	34	18%
	SI	2,15	−2,85	5	20	125	16%
SN	SNc	−4,6	−6,5	1,9	6	48	13%
	SNr	−4,45	−6,65	2,2	8	55	15%
VTA	VTA	−4,45	−7,1	0,95	8	66	12%
STN	STN	−3,25	−4,2	2,65	4	24	17%

The basal ganglia regions in the *Brain Maps* (version 3) are listed with their abbreviation under Abb.) and sorted into broad hierarchical groupings as defined in the main paper. The table gives the rostral and caudal end of each structure relative to Bregma, extracted from the atlas plates*. The total length was estimated as the distance (in mm) between the rostral and caudal end. The number of sampled sections are given, and corresponds to the number of atlas plates containing the region annotation. The total number of sections was calculated based on the total length and the section thickness (40 µm). Lastly, the sample ratio gives the ratio of sampled sections to estimated total number of sections. *Note that the Bregma levels are extracted from version 1 of the atlas, as this was the only hard copy we had available, and these are not included in the digital versions.

**Fig. 7:**
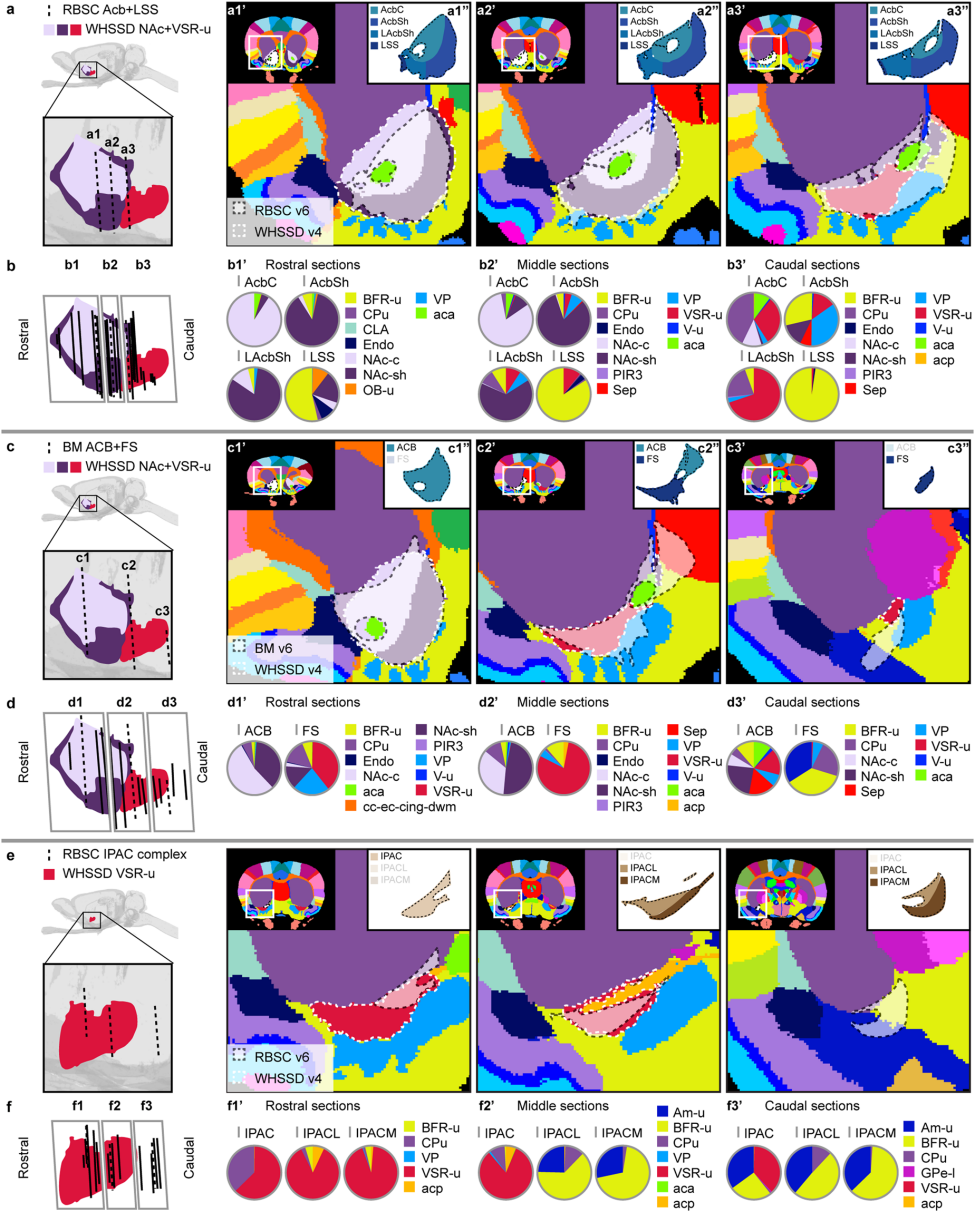
Quantitative spatial analysis of the nucleus accumbens (Acb/ACB) and other ventral striatal regions across rat brain atlases. (**a**) Lateral view of the *WHS rat brain atlas (WHSSD)* NAc (NAc-sh, dark purple; NAc-c, light purple) and VSR-u (red), with magnified panel showing the spatial position and angle of three selected atlas plates (dashed lines) from rostral (a1), middle (a2) and caudal (a3) parts of *The Rat Brain in Stereotaxic Coordinates (RBSC)* Acb and LSS. The *WHSSD* cut to correspond with the three selected atlas plates from *RBSC* (a1’-a3’), showing the spatial correspondence of the Acb and LSS (black dashed line filled with a transparent white colour) with regions in the *WHSSD*. The *WHSSD* NAc and VSR-u is highlighted with white dashed line for comparison. The inset (a1”-a3”) show the LSS and the subdivisions of the Acb. (**b**) Lateral view of the *WHSSD* NAc and VSR-u, showing the spatial position and angle of the selected atlas plates (**a1-a3**, dashed lines) and the remaining *RBSC* atlas plates including Acb and LSS annotations (solid lines). The boxes (b1-b3) indicate the three rostro-caudal parts used for analysis of the quantitative spatial overlap of the *RBSC* Acb and LSS with regions in the *WHSSD*. The pie charts (b1’-b3’) show the proportion of the *RBSC* LSS and Acb subdivisions situated in regions of the *WHSSD*. (**c,d**) Selected atlas plates (c1-c3,c1’-c3’,c1”-c3”), spatial position (d1-d3) and pie charts (d1’-d3’) for the *Brain Maps (BM)* ACB and FS annotations in relation to *WHSSD* regions, as described for (**a,b**). **e-f**) Selected atlas plates (e1-e3,e1’-e3’,e1”-e3”), spatial position (f1-f3) and pie charts (f1’-f3’) for the *RBSC* IPAC complex annotation in relation to *WHSSD* regions, as described for (**a,b**). Note that the atlas plates from *BM* are registered with a mediolateral angle of 4 degrees, which in c1-c3 and d1-d3 gives an illusion of a dorsoventral angle in some atlas plates. **Abbreviations:** aca, anterior commissure, anterior limb; aca-p, anterior commissure, posterior limb; ACB, nucleus accumbens; AcbC, accumbens nucleus, core; AcbSh, accumbens nucleus, shell; Am-u, amygdaloid area, unspecified; BFR-u, basal forebrain region, unspecified; BM, *Brain Maps*; CPu, caudate putamen; CLA, claustrum; Endo, endopiriform nucleus; GPe-l, globus pallidus external, lateral part; LAcbSh, lateral accumbens shell; LSS, lateral stripe of striatum; NAc, nucleus accumbens; NAc-c, nucleus accumbens, core; NAc-sh, nucleus accumbens, shell; OB-u, olfactory bulb, unspecified; PIR3; piriform cortex, layer 3; RBSC, *The Rat Brain in Stereotaxic Coordinates*; Sep, septal region; VSR-u, ventral striatal region, unspecified; V-u, ventricular system, unspecified; VP, ventral pallidum; WHSSD, *WHS rat brain atlas*.

**Fig. 8:**
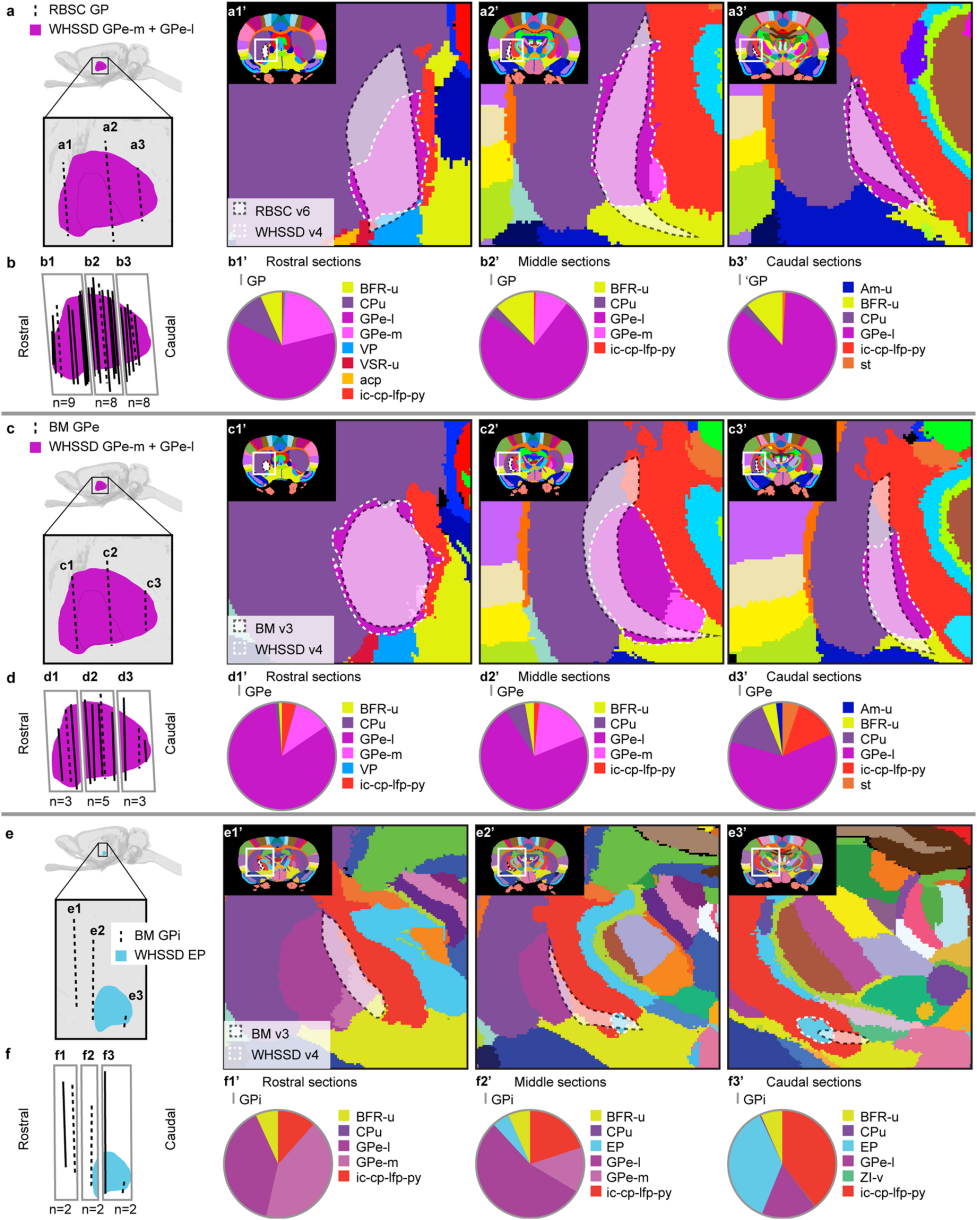
Quantitative spatial analysis of the globus pallidus external (GPe) and internal (GPi) across rat brain atlases. (**a**) Lateral view of the *WHS rat brain atlas (WHSSD)* GPe (pink), with magnified panel showing the spatial position and angle of three selected atlas plates (dashed lines) from rostral (a1), middle (a2) and caudal (a3) part of *The Rat Brain in Stereotaxic Coordinates (RBSC)* GPe. The *WHSSD* cut to correspond with the three selected atlas plates from *RBSC* (a1’-a3’), showing the spatial correspondence of the GPe (black dashed line filled with a transparent white colour) with regions in the *WHSSD*. The *WHSSD* GPe-m and GPe-l are highlighted for comparison (white dashed lines). (**b**) Lateral view of the *WHSSD* GPe, showing the spatial position and angle of the selected atlas plates (a1-a3, dashed lines**)** and the remaining *RBSC* atlas plates including GPe annotations (solid lines). The boxes (b1-b3) indicate the three rostro-caudal parts used for analysis of the quantitative spatial overlap of the *RBSC* GPe with regions in the *WHSSD*. The pie charts (b1’-b3’) show the proportion of the *RBSC* GPe annotation situated in regions of the *WHSSD*. (**c,d**) Selected atlas plates (c1-c3,c1’-c3’), spatial position (d1-d3) and pie charts (d1’-d3’) for the *Brain Maps (BM)* GPe annotation in relation to *WHSSD* regions, as described for (**a,b**). (**e,f**) Selected atlas plates (e1-e3,e1’-e3’), spatial position (f1-f3) and pie charts (f1’-f3’) for the *BM* GPi annotation in relation to *WHSSD* regions, as described for (**a,b**). Note that the atlas plates from *BM* are registered with a mediolateral angle of 4 degrees, which in c1-c3, d1-d3,e1-e3 and f1-f3 gives an illusion of a dorsoventral angle in some atlas plates. **Abbreviations:** aca-p, anterior commissure, posterior limb; Am-u, amygdaloid area, unspecified; BFR-u, basal forebrain region, unspecified; BM, *Brain Maps*; CPu, caudate putamen; EP, entopeduncular nucleus; GP, globus pallidus; GPe, globus pallidus external; GPi, globus pallidus internal; GPe-l, globus pallidus external, lateral part; GPe-m, globus pallidus external, medial part; ic-cp-lfp-py, corticofugal tract and corona radiata; VP, ventral pallidum; VSR-u, ventral striatal region, unspecified; RBSC, *The Rat Brain in Stereotaxic Coordinates*; st, stria terminalis; WHSSD, *WHS rat brain atlas;* ZI-v, zona incerta, ventral part.

**Fig. 9:**
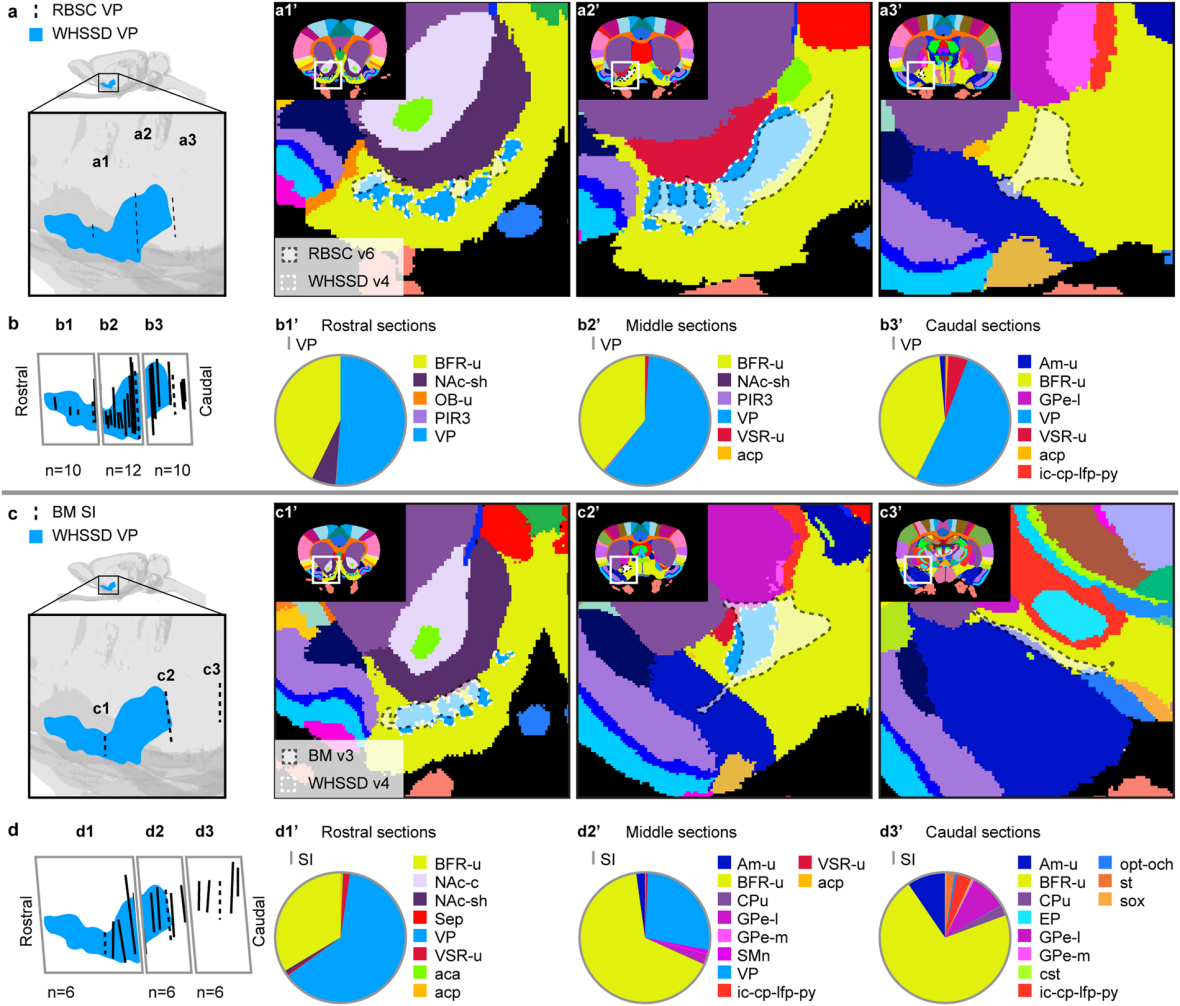
Quantitative spatial analysis of the ventral pallidum (VP) and substantia innominata (SI) across rat brain atlases. (**a**) Lateral view of the *WHS rat brain atlas (WHSSD)* VP (light blue), with magnified panel showing the spatial position and angle of three selected atlas plates (dashed lines) from rostral (a1), middle (a2) and caudal (a3) parts of *The Rat Brain in Stereotaxic Coordinates (RBSC)* VP. The *WHSSD* cut to correspond with the three selected atlas plates from *RBSC* (a1’-a3’), showing the spatial correspondence of the VP (black dashed line filled with a transparent white colour) with regions in the *WHSSD*. The *WHSSD* VP is highlighted for comparison (white dashed lines). (**b**) Lateral view of the *WHSSD* VP, showing the spatial position and angle of the selected atlas plates (a1-a3, dashed lines**)** and the remaining *RBSC* atlas plates including VP annotations (solid lines). The boxes (b1-b3) indicate the three rostro-caudal parts used for analysis of the quantitative spatial overlap of the *RBSC* VP with regions in the *WHSSD*. The pie charts (b1’-b3’) show the proportion of the *RBSC* VP annotation situated in regions of the *WHSSD*. (**c,d**) Selected atlas plates (c1-c3,c1’-c3’), spatial position (d1-d3) and pie charts (d1’-d3’) for the *Brain Maps (BM)* SI annotation in relation to *WHSSD* regions, as described for (**a,b**). Note that the atlas plates from *BM* are registered with a mediolateral angle of 4 degrees, which in c1-c3 and d1-d3 gives an illusion of a dorsoventral angle in some atlas plates. **Abbreviations:** aca, anterior commissure, anterior limb; acp, anterior commissure, posterior limb; AM-u, amygdaloid area, unspecified; BFR-u, basal forebrain region, unspecified; BM, *Brain Maps*; CPu, caudate putamen; cst, commisural stria terminalis; EP, entopeduncular nucleus; GPe-l, globus pallidus external, lateral part; GPe-m, globus pallidus external, medial part; ic-cp-lfp-py, corticofugal tract and corona radiata; NAc-c, nucleus accumbens, core; NAc-sh, nucleus accumbens, shell; OB-u, olfactory bulb, unspecified; opt-och, optic tract and optic chiasm; PIR3, piriform cortex, layer 3; RBSC, *The Rat Brain in Stereotaxic Coordinates*; Sep, septral region; SI, innominate substance; SMn, nucleus of the stria medullaris; st, stria terminalis; sox, supraoptic decussation; VP, ventral pallidum; VSR-u, ventral striatal region, unspecified; WHSSD, *WHS rat brain atlas*.

**Fig. 10:**
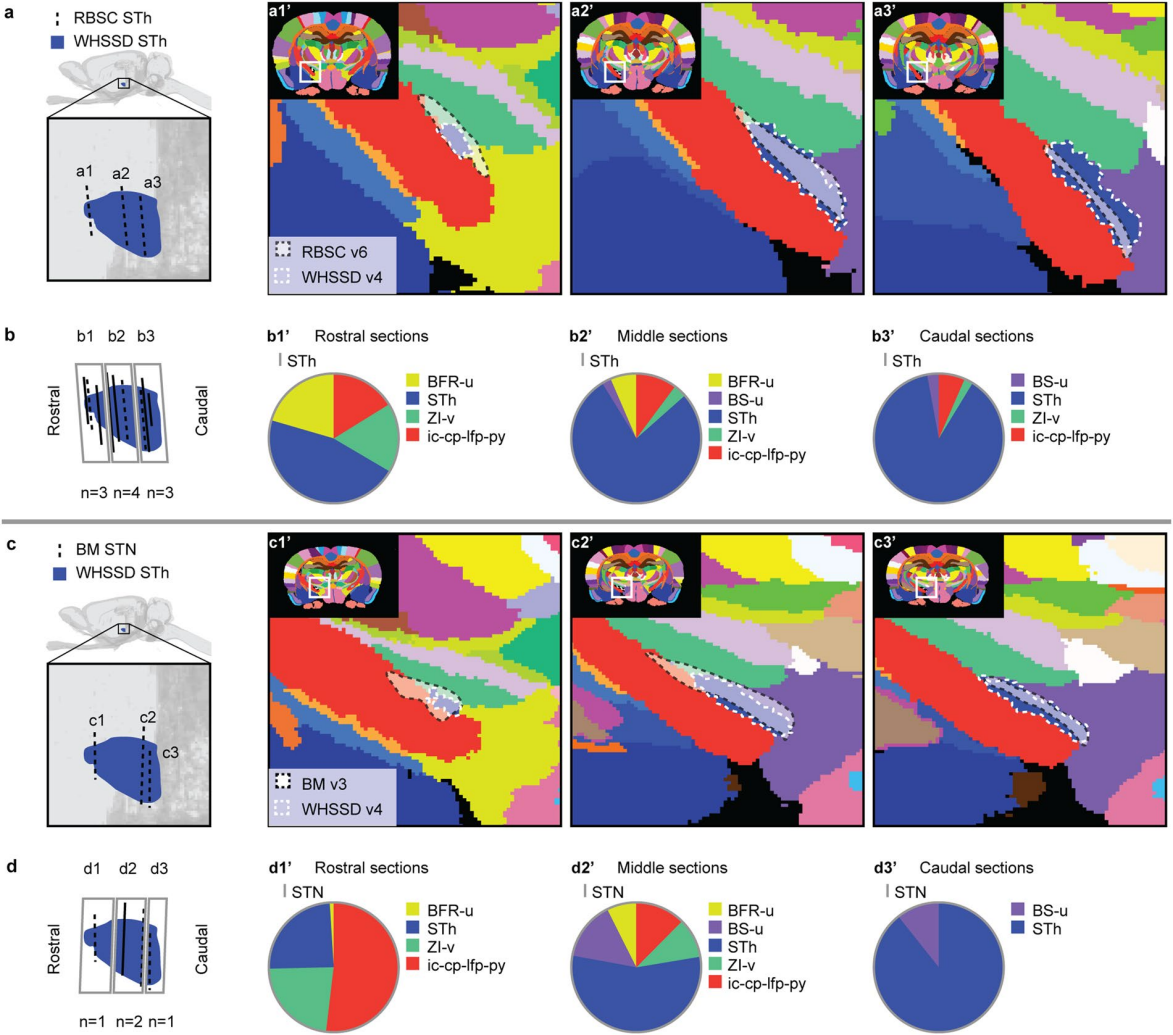
Quantitative spatial analysis of the subthalamic nucleus (STh/STN) across rat brain atlases. (**a**) Lateral view of the *WHS rat brain atlas (WHSSD)* STh (blue), with magnified panel showing the spatial position and angle of three selected atlas plates (dashed lines) from rostral (a1), middle (a2) and caudal (a3) part of *The Rat Brain in Stereotaxic Coordinates (RBSC)* STh. The *WHSSD* cut to correspond with the three selected atlas plates from *RBSC* (a1’-a3’), showing the spatial correspondence of the STh (black dashed line filled with a transparent white colour) with regions in the *WHSSD*. The *WHSSD* STh is highlighted for comparison (white dashed lines). (**b**) Lateral view of the *WHSSD* STh, showing the spatial position and angle of the selected atlas plates (a1-a3, dashed lines) and the remaining *RBSC* atlas plates including STh annotations (solid lines). The boxes (b1-b3) indicate the three rostro-caudal parts used for analysis of the quantitative spatial overlap of the *RBSC* STh with regions in the *WHSSD*. The pie charts (b1’-b3’) show the proportion of the *RBSC* STh annotation situated in regions of the *WHSSD*. (**c,d**) Selected atlas plates (c1-c3,c1’-c3’), spatial position (d1-d3) and pie charts (d1’-d3’) for the *Brain Maps (BM)* STN annotation in relation to *WHSSD* regions, as described for (**a,b**). Note that the atlas plates from *BM* are registered with a mediolateral angle of 4 degrees, which in c1-c3 and d1-d3 gives an illusion of a dorsoventral angle in some atlas plates. **Abbreviations:** BM, *Brain Maps*; BFR-u, basal forebrain region, unspecified; BS-u, brainstem, unspecified; ic-cp-lfp-py, corticofugal tract and corona radiata; RBSC, *The Rat Brain in Stereotaxic Coordinates*; STh/STN, subthalamic nucleus; WHSSD, *WHS rat brain atlas*; ZI-v, zona incerta, ventral part.

**Fig. 11:**
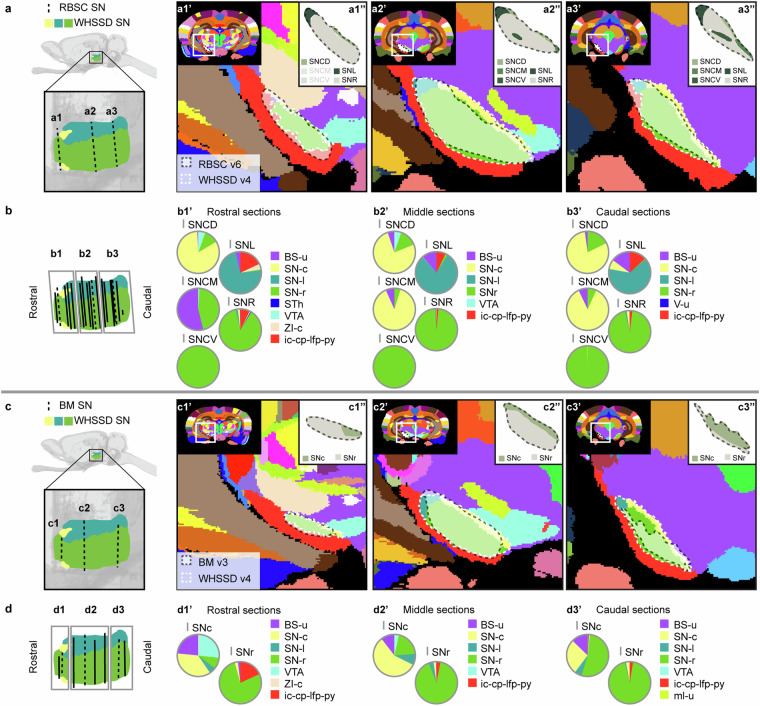
Quantitative spatial analysis of the substantia nigra (SN) and its subdivisions across rat brain atlases. (**a**) Lateral view of the *WHS rat brain atlas (WHSSD)* SN (SN-c, yellow; SN-l, petrol green; SN-r, light green), with magnified panel showing the spatial position and angle of three selected atlas plates (dashed lines) from rostral (a1), middle (a2) and caudal (a3) part of *The Rat Brain in Stereotaxic Coordinates (RBSC)* SN. The *WHSSD* cut to correspond with the three selected atlas plates from *RBSC* (a1’-a3’), showing the spatial correspondence of the SN annotations (black dashed line filled with a transparent white colour) with regions in the *WHSSD*. The *WHSSD* SN is highlighted for comparison (white dashed lines). The inset (a1”-a3”) shows the subdivisions of the SN. (**b**) Lateral view of the *WHSSD* SN, showing the spatial position and angle of the selected atlas plates (a1-a3, dashed lines) and the remaining *RBSC* atlas plates including SN annotations (solid lines). The boxes (b1-b3) indicate the three rostro-caudal parts used for analysis of the quantitative spatial overlap of the *RBSC* SN with regions in the *WHSSD*. The pie charts (b1’-b3’) show the proportion of the *RBSC* SN subdivisions situated in regions of the *WHSSD*. (**c,d**) Selected atlas plates (c1-c3,c1’-c3’,c1”-c3”), spatial position (d1-d3) and pie charts (d1’-d3’) for the *Brain Maps (BM)* SN annotations in relation to *WHSSD* regions, as described for (**a,b**). Note that the atlas plates from *BM* are registered with a mediolateral angle of 4 degrees, which in c1-c3 and d1-d3 gives an illusion of a dorsoventral angle in some atlas plates. **Abbreviations:** BM, *Brain Maps*; BS-u, brainstem, unspecified; ic-cp-lfp-py, corticofugal tract and corona radiata; ml-u, medial lemniscus, unspecified; RBSC, *The Rat Brain in Stereotaxic Coordinates*; SN, substantia nigra; SNc/SN-c, substantia nigra, compact part; SNCD, substantia nigra, compact part, dorsal tier; SNCM, substantia nigra, compact part, medial tier; SNCV, substantia nigra, compact part, ventral tier; SNL/ SN-l, substantia nigra, lateral part; SNR/SNr/ SN-r, substantia nigra, reticular part; STh, subthalamic nucleus; V-u, ventricular system, unspecified; VTA, ventral tegmental area; WHSSD, *WHS rat brain atlas*; ZI-c, zona incerta, caudal part.

**Fig. 12:**
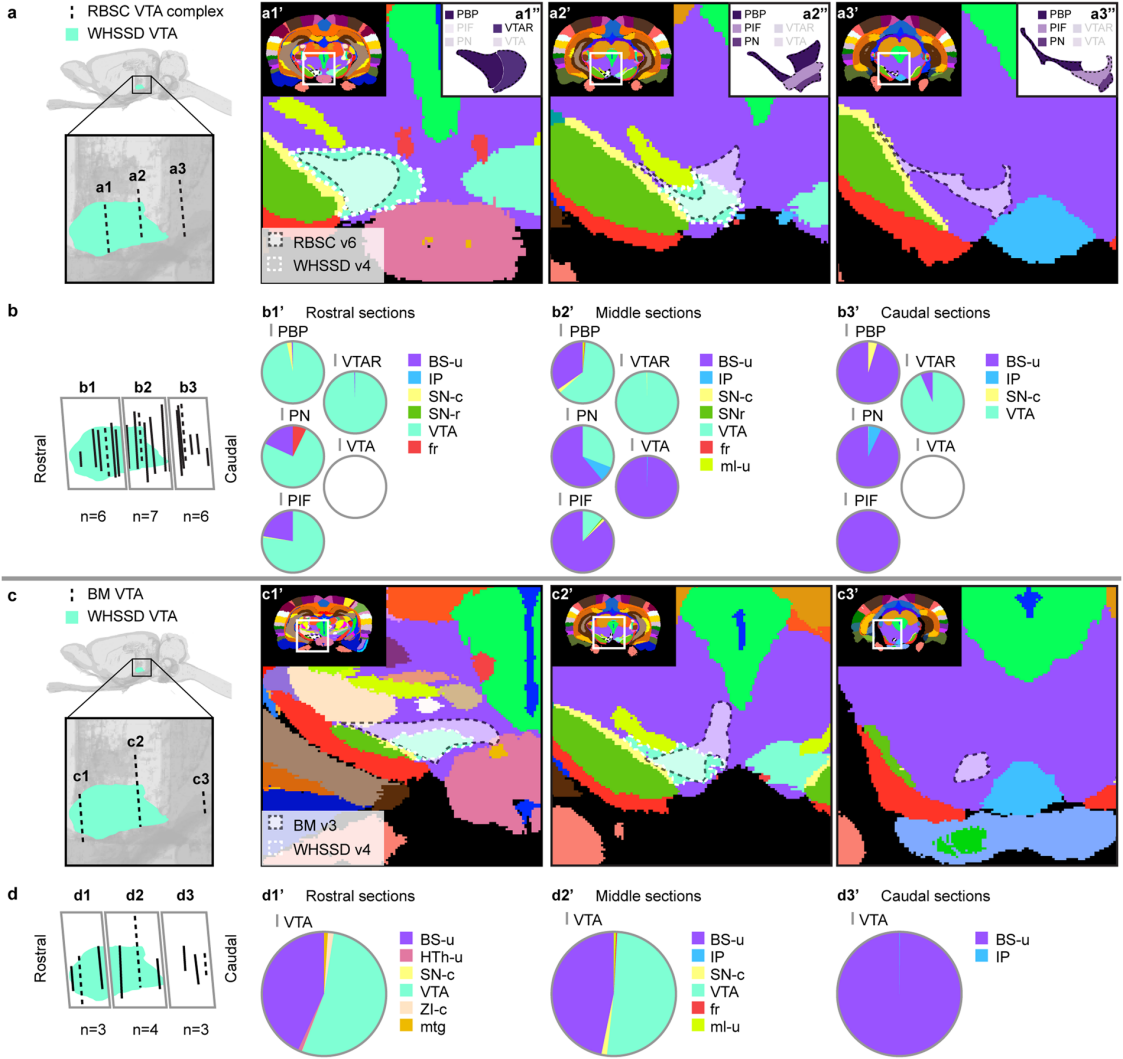
Quantitative spatial analysis of the ventral tegmental area (VTA) and its subdivisions across rat brain atlases. (**a**) Lateral view of the *WHS rat brain atlas (WHSSD)* VTA (mint green), with magnified panel showing the spatial position and angle of three selected atlas plates from rostral (a1), middle (a2) and caudal (a3) part of *The Rat Brain in Stereotaxic Coordinates (RBSC)* VTA complex. The *WHSSD* cut to correspond with the three selected atlas plates from *RBSC*, (a1’-a3’) show the spatial correspondence of the VTA complex annotations (black dashed line filled with a transparent white colour) with regions in the *WHSSD*. The *WHSSD* VTA is highlighted for comparison (white dashed lines). The inset (a1”-a3”) shows the subdivisions of the VTA complex. (**b**) Lateral view of the *WHSSD* VTA, showing the spatial position and angle of the selected atlas plates (a1-a3, dashed lines) and the remaining *RBSC* atlas plates including VTA complex annotations (solid lines). The boxes (b1-b3) indicate the three rostro-caudal parts used for analysis of the quantitative spatial overlap of the *RBSC* VTA complex with regions in the *WHSSD*. The pie charts (b1’-b3’) show the proportion of the *RBSC* VTA complex subdivisions situated in regions of the *WHSSD*. (**c,d**) Selected atlas plates (c1-c3,c1’-c3’), spatial position (d1-d3) and pie charts (d1’-d3’) for the *Brain Maps (BM)* VTA annotations in relation to *WHSSD atlas* regions, as described for (**a,b**). Note that the atlas plates from *BM* are registered with a mediolateral angle of 4 degrees, which in c1-c3 and d1-d3 gives an illusion of a dorsoventral angle in some atlas plates. **Abbreviations:** BM, *Brain Maps*; BS-u, brainstem, unspecified; fr, fasciculus retroflexus; HTh-u, hypothalamic region, unspecified; IP, interpeduncular nucleus; ml-u, medial lemniscus, unspecified; mtg, mammillotegmental tract; PBP, parabrachial pigmented nucleus of the VTA; PIF, parainterfascicular nucleus of the VTA; PN, paranigral nucleus of the VTA; RBSC, *The Rat Brain in Stereotaxic Coordinates*; SN-c, substantia nigra, compact part; SN-r, substantia nigra, reticular part; VTA, ventral tegmental area; VTAR, ventral tegmental area, rostral part; WHSSD, *WHS rat brain atlas*; ZI-c, zona incerta, caudal part.

Figures 6–12 visualise the qualitative and quantitative analyses of spatial overlap for basal ganglia regions across atlases. Each figure consists of two or three paired panels showing the qualitative and quantitative overlap of one basal ganglia region in either *The Rat Brain in Stereotaxic Coordinates* or *Brain Maps* with regions in the *WHS rat brain atlas*. The top row in each paired panel shows the spatial overlap of the region in three selected atlas plates from rostral to caudal. The row of pie charts below shows the quantitative data based on all available atlas plates grouped into rostral, middle, and caudal sections. In some cases, multiple subregions are represented in one paired panel (e.g. Figure 7a,b, which includes all ventral striatal subregions). In these cases, the atlas plates in the top panels are selected to provide samples through all subregions and to visualise key observations. However, due to different rostrocaudal extents of the individual subregions, these sections might not represent the rostral, middle, and caudal thirds of each subregion. For example, the image in Figure 7a3’ is from the caudal part of the accumbens nucleus core, accumbens nucleus shell, and lateral accumbens shell, but from the middle part of the lateral stripe of striatum. Figures 6–12 are closely related to the textual descriptions of the spatial relationships and are intended to support the reader in interpreting and visualising these.

### Caudate-putamen

The **caudate-putamen (CPu**; Fig. 6**)** appears across all the atlases investigated, referred to as “caudate putamen (striatum)” (CPu) in *The Rat Brain in Stereotaxic Coordinates* (Table 1; represented in Fig. 6a,b), and “caudoputamen” (CP) in *Brain Maps* (Table 2; represented in Fig. 6c,d). This region is very similar across atlases. Quantitatively, 96% of *The Rat Brain in Stereotaxic Coordinates* CPu is situated in the *WHS rat brain atlas* CPu (Fig. 6b1’-3’). The remaining 4% of the region are located across 19 regions in *WHS rat brain atlas*. For the *Brain Maps* CP, 94% is situated within the CPu in *WHS rat brain atlas*, while the remaining 6% is located across 20 regions, mostly located ventrally (Fig. 6d1’-3’).

Qualitatively, the dorsal and lateral borders of the CPu with the corpus callosum are very similar between the three atlases (Fig. 6a1’-3’,c1’-3’). The ventral border of the CPu varies across atlases, and we therefore focused our comparison on this border. The ventral CPu border is with the nucleus accumbens (NAc) rostrally, and the anterior commissure and various basal forebrain regions caudally. In *The Rat Brain in Stereotaxic Coordinates*, the CPu-NAc border appears more ventrally than in the *WHS rat brain atlas*, extending at a ~45° angle from the tip of the lateral ventricle (Fig. 6a1’). This boundary curves outwards from a slightly more dorsal point on the ventricle in the *WHS rat brain atlas*. In *Brain Maps*, this CP-ACB border curves dorsally in some atlas plates and ventrally in others (e.g. the one shown in Fig. 6c1’), thus appearing similar to the corresponding border in *WHS rat brain atlas* in some atlas plates and different in others. This highlights the difficulty in distinguishing the CP-ACB border and explains the discrepancies in the quantitative analysis.

Caudal to the nucleus accumbens in *The Rat Brain in Stereotaxic Coordinates*, the CPu extends further ventrally than in preceding and following atlas plates (see asterisk in Fig. 6b1). This area corresponds to the ventral striatal region, unspecified (VSR-u) in the *WHS rat brain atlas* and likely represents a zone of transition between different ventral striatal regions that is challenging to define consistently. At mid- to caudal levels of the CPu in *The Rat Brain in Stereotaxic Coordinates*, the ventral border is with the posterior interstitial limb of the anterior commissure (IPACL), and this border is very similar to the *WHS rat brain atlas* border (Fig. 6a2’-3’; but see “Overview of basal ganglia region terms and definitions across atlas versions” for important changes to this across *The Rat Brain in Stereotaxic Coordinates*). In *Brain Maps*, the middle part of CP borders the striatal fundus ventrally, largely resembling the CPu boundary in the *WHS rat brain atlas* (Fig. 6c2’). In *The Rat Brain in Stereotaxic Coordinates*, the ventral border of the CPu is not defined at caudal levels (Fig. 6c3’). In the *WHS rat brain atlas*, this border is with the amygdaloid area, unspecified, which explains its overlap with the CPu in both atlases in the quantitative analysis (Fig. 6b3’,d3’).

### Nucleus accumbens and other ventral regions of the striatum

We found that 73% of the **nucleus accumbens, core (AcbC**; Fig. 7) in *The Rat Brain in Stereotaxic Coordinates* (represented in Fig. 7a,b) is located within the NAc-c annotated in the *WHS rat brain atlas*. The remaining parts of NAc-c in *The Rat Brain in Stereotaxic Coordinates* NAc-c are situated across 11 *WHS rat brain atlas* regions, including CPu (8.51%), NAc-sh (6.30%), anterior commissure, anterior limb (5.38%) and ventral striatal regions, unspecified (5.28%) (see Fig. 7b1’-3’). The rostral part of the core is very similar in the two atlases (Fig. 6a1’,c1’), although the border with the CPu seems to be defined more ventrally in *The Rat Brain in Stereotaxic Coordinates*. Caudally, the NAc-c also looks very similar in the two atlases, although the region extends more caudally in *The Rat Brain in Stereotaxic Coordinates*, and this caudal part is situated mainly in the CPu and the VSR-u in *WHS rat brain atlas* (Fig. 6a3’,b3’). For *The Rat Brain in Stereotaxic Coordinates* AcbSh, 69% is situated in the *WHS rat brain atlas* NAc-sh. The remaining parts are situated across 11 regions in the *WHS rat brain atlas*, mainly the ventral pallidum (12%), basal forebrain region, unspecified (10%), and ventral striatal region, unspecified (5.26%) (see Fig. 7b1’-3’). The rostral extent of the NAc-sh is similar in the two atlases, and it is mainly the caudal part of *The Rat Brain in Stereotaxic Coordinates* AcbSh that has discrepancies with the *WHS rat brain atlas* (Fig. 7a3’,a3”,b3,b3’). This is due to the larger caudal extent of the AcbSh in *The Rat Brain in Stereotaxic Coordinates*. There are also differences between the two due to the LAcbSh in *The Rat Brain in Stereotaxic Coordinates*, which makes up the lateral part of the *WHS rat brain atlas* NAc-sh (see below for details).

In *Brain Maps* (represented in Fig. 7c,d), the nucleus accumbens (ACB) is delineated as one region without subdivisions. Quantitatively, 33% and 41% of the Swanson ACB is situated in the NAc-c and NAc-sh of the *WHS rat brain atlas*, respectively (Fig. 7d1’-3’). The remaining *Brain Maps* ACB is located across nine regions in the *WHS rat brain atlas*, mainly the CPu (8.74%). The discrepancies are largest caudally, where *Brain Maps* ACB extends dorsally and medially into areas of the *WHS rat brain atlas* septal and basal forebrain regions (Fig. 7c2’,c2”,d3’). The *Brain Maps* ACB can thus be considered to include both the NAc-c and NAc-sh in the *WHS rat brain atlas*.

The LAcbSh in *The Rat Brain in Stereotaxic Coordinates* is described as “resembling the CPu in some ways and the NAc-sh in others”. Indeed, our quantitative analysis showed that 40% and 12% of the LAcbSh (represented in Fig. 7a,b) is situated in the NAc-sh and CPu, respectively. However, a large part of it is also situated in the VSR-u (34%), and a smaller part (6.35%) is located in the basal forebrain region of the *WHS rat brain atlas*. The LAcbSh can therefore be considered to overlap with the *WHS rat brain atlas* NAc-sh rostrally and VSR-u caudally (Fig. 7a1’,a1”,b1’,a3’,a3”,b3’, respectively).

The IPAC in *The Rat Brain in Stereotaxic Coordinates* (represented in Fig. 7e,f) is mainly situated in the VSR-u (67.45%), followed by the CPu (12%), the amygdaloid area, unspecified (8.90%), and the basal forebrain region, unspecified (6.97%) (see Fig. 7f1’-3’). The IPACL and IPACM (Fig. 7e1”-3”) are situated caudally in the IPAC complex (Fig. 7e1’-3’) and are mainly located in the *WHS rat brain atlas* basal forebrain region, unspecified (41 and 46%, respectively) followed by the VSR-u (25 and 29%, respectively). Their rostral parts are almost completely within the VSR-u (Fig. 7e2’,e2”,f1’), whereas their caudal parts are mainly situated in the basal forebrain region, unspecified (Fig. 7e3’,e3”,f3’). This corroborated our qualitative observations that the rostral parts of the IPAC complex overlaps with the VSR-u but extends far more caudally into the *WHS rat brain atlas* basal forebrain region.

We found that most of the LSS (76%) from *The Rat Brain in Stereotaxic Coordinates* (represented in Fig. 7a,b) is situated in the *WHS rat brain atlas* basal forebrain region, unspecified (BFR-u); the remaining overlap was primarily with the NAc-sh (8%). The most rostral part of the LSS is embedded inside the *WHS rat brain atlas* NAc-sh, but the extent of this overlap is very small when considering its long rostrocaudal extent, so that even the rostral segment of the LSS only has a small portion located within the NAc-sh (Fig. 7b1’). For most of its extent, the LSS is situated in a dorsal extension of the *WHS rat brain atlas* BFR-u wedged in between the dorsal endopiriform nucleus and the VSR-u (Fig. 7b2’-3’).

*Brain Maps* include a ventral striatal region caudal to the ACB referred to as the striatal fundus (FS; represented in Fig. 7c,d). This region is mainly situated in the *WHS rat brain atlas* VSR-u (44%). The remaining parts are located across ten *WHS rat brain atlas* regions, including the VP (14%), basal forebrain region, unspecified (13%), CPu (12%), and NAc-sh (7.28%) (see Fig. 7d1’-3’). The FS in *Brain Maps* overlaps partly with the CPu and partly with the NAc-sh in *WHS rat brain atlas* at the rostral extreme (consistent with *The Rat Brain in Stereotaxic Coordinates* description of this term; see above), but the remainder of the rostral and middle part of the FS is situated mainly in the VSR-u (Fig. 7c2’,c2”,d1’-d2’). The caudal part of the FS is located in the zone of transition between the CPu and the BFR-u and amygdaloid areas (Fig. 7c3’,c3”,d3’).

### Globus pallidus external and entopeduncular nucleus

We found that 70% of the **globus pallidus external (GP**; Fig. 8**)** from *The Rat Brain in Stereotaxic Coordinates* (represented in Fig. 8a,b) is situated in the *WHS rat brain atlas* GPe-l, while 13% is situated in the GPe-m. The remaining parts are located across eight regions, mainly the BF-u (9.52%) and CPu (6.59%) (see Fig. 8b1’-3’). The GP in *The Rat Brain in Stereotaxic Coordinates* includes both the GPe-l and GPe-m of the *WHS rat brain atlas*, with the GPe-m only appearing at middle levels of *The Rat Brain in Stereotaxic Coordinates* GP (Fig. 8a2’). The GP is, however, larger than the combination of these two regions from the *WHS rat brain atlas*: the rostral part has a larger dorsal extent (Fig. 8a1’), whereas the middle and caudal parts have a larger ventral extent (Fig. 8a2’-3’). These differences explain the quantitative overlap with the BFR-u and CPu. The larger ventral extent of *The Rat Brain in Stereotaxic Coordinates* GP is partly accounted for by the basal nucleus of Meynert, which is included without borders in their atlas and was therefore included in the GP annotation in the current analysis. The **entopeduncular nucleus (EP)** in *The Rat Brain in Stereotaxic Coordinates* is mainly situated in the *WHS rat brain atlas* EP (93%), corroborating our qualitative assessment that these regions are identical. Small discrepancies are due to differences in the borders between the EP and surrounding white matter.

The GPe in *Brain Maps* (represented in Fig. 8c,d) is situated mainly within the *WHS rat brain atlas* GPe-l (74%) and GPE-m (14%). The remaining parts are situated across six regions of the *WHS rat brain atlas*, with the most substantial part located in the *WHS rat brain atlas* CPu (5.46%) (see Fig. 8d1’-3’). The rostral part of the GPe in *Brain Maps* is very similar to the *WHS rat brain atlas* GPe-l (Fig. 7c1’), with the only difference being that the *WHS rat brain atlas* region appears bigger at the rostral extreme. The *Brain Maps* GPe is not subdivided, thus it also overlaps with the GPe-m in the *WHS rat brain atlas* at middle levels (Fig. 7c2’). In the middle and caudal parts, the *Brain Maps* GPe has a substantially larger dorsal extent than the GPe-m in the *WHS rat brain atlas* (Fig. 7c3’). Thus, the *Brain Maps* GPe overlaps with both the GPe-m and GPe-l in the *WHS rat brain atlas*, mostly with the lateral part; however, it does not include either of them fully. This is due to the annotation of the *Brain Maps* GPi, (represented in Fig. 8e,f), which curiously overlaps with both the GPe-m, GPe-l and EP of the *WHS rat brain atlas*. The largest part of the *Brain Maps* GPi is situated in the GPe-l (38%), followed by the corticofugal tract and corona radiata (23%), GPe-m (20%) and EP (13%) (see Fig. 8f1’-3’). The rostral part of *Brain Maps* GPi overlaps with the GPe-l dorsolaterally and GPe-m ventromedially (Fig. 8e1’), after which it extends gradually into the internal capsule as a medial continuation of the GPe (Fig. 8e2’). At caudal levels, *Brain Maps* GPi is embedded in the external capsule, but is more elongated in shape than the *WHS rat brain atlas* EP (Fig. 8e3’). As such, *Brain Maps* GPe resembles the EP in some levels and the GPe-m in others but cannot be said to correspond to either one of them.

### Ventral pallidum

In our quantitative analysis, we found that 56% of the **ventral pallidum (VP**; Fig. 9**)** in *The Rat Brain in Stereotaxic Coordinates* (represented in Fig. 9a,b) is situated in the *WHS rat brain atlas* VP, while 40% is situated in the basal forebrain region, unspecified (Fig. 9b1’-3’). Rostrally, the VP in both atlases extend as finger-like protrusions and appear in the coronal plane as small circular islands embedded in the basal forebrain region; these islands overlap but are not perfectly aligned (Fig. 9a1’). The VP in the *WHS rat brain atlas* starts extending dorsally and medially earlier than *The Rat Brain in Stereotaxic Coordinates* VP. More caudally, the VP in *The Rat Brain in Stereotaxic Coordinates* seems slightly more extensive (Fig. 9a2’). It also extends further caudally than the *WHS rat brain atlas* VP, with its caudal part situated largely in the *WHS rat brain atlas* basal forebrain (Fig. 9a3’,b3’). Thus, the VP in *The Rat Brain in Stereotaxic Coordinates* can be considered to overlap with the VP in the *WHS rat brain atlas*. *The Rat Brain in Stereotaxic Coordinates* use the term “substantia innominata, basal part” (SIB) for the parts that remain after delineating the VP; this region does not overlap with any basal ganglia regions in *WHS rat brain atlas* and was therefore not included in the quantitative analysis.

In *Brain Maps*, the VP is not delineated separately; instead, there is a large collective area referred to as the substantia innominata (SI) (represented in Fig. 9c,d). The SI in *Brain Maps* is mainly situated in the *WHS rat brain atlas* BFR-u (56%) and VP (33%) (see Fig. 9d1’-3’). The remaining parts of the *Brain Maps* SI is spread across 17 regions (see Table 3). The rostral and middle parts overlap with the VP in the *WHS rat brain atlas* (Fig. 9d1’-2’) but has a larger medial extent that overlaps with the *WHS rat brain atlas* basal forebrain, unspecified (Fig. 9c1’-2’). The caudal part is situated mostly in the *WHS rat brain atlas* BFR-u, with some parts overlapping the amygdaloid area (Fig. 9c3’,d3’).

### Subthalamic nucleus

The **subthalamic nucleus (STh**; Fig. 10) is present in all the brain atlases surveyed here and both the terms and their respective borders appear similar across all versions of each atlas (Tables 1, 2). Despite this, our quantitative analysis (Fig. 10a–d) showed that the STh was less similar across atlases than expected, with 70% of the STh in *The Rat Brain in Stereotaxic Coordinates* (represented in Fig. 10a,b) and 55% of the STN in *Brain Maps* (represented in Fig. 10c,d) situated in the *WHS rat brain atlas* STh (see Table 3). The STh in *The Rat Brain in Stereotaxic Coordinates* is generally similar to the *WHS rat brain atlas* STh but appears larger rostrally (Fig. 10a1’,b1’) and smaller caudally (Fig. 10a3’). The *Brain Maps* STN generally appears slightly bigger than the *WHS rat brain atlas* STh (Fig. 10c1’-3’), especially rostrally, where the quantitative overlap is also smallest (Fig. 10d1’).

### Substantia nigra

For the **substantia nigra (SN**; Fig. 11), compact part, dorsal tier (SNCD; Fig. 11a1”-3”) in *The Rat Brain in Stereotaxic Coordinates* (represented in Fig. 11a,b), we found that 80% is situated within the SNc, 13% within the SNr, and 4.46% within the VTA of the *WHS rat brain atlas*. The substantia nigra, compact part, medial tier (SNCM; Fig. 11a2”-3”) is also mainly situated in the *WHS rat brain atlas* SNc (80%), followed by the brainstem, unspecified (11%) and the SNr (9%). Qualitatively, the combined SNCD and SNCM in *The Rat Brain in Stereotaxic Coordinates* is very similar to the *WHS rat brain atlas* SN-c, with the SNCD making up the largest part (Fig. 11a1”-3”). The substantia nigra, compact part, ventral tier (SNCV; Fig. 11a2’-3’) in *The Rat Brain in Stereotaxic Coordinates* is embedded inside the reticular part, which means that it is part of the SN-r in *WHS rat brain atlas*. Quantitatively, it is situated completely (94%) within the *WHS rat brain atlas* SNr (Fig. 11b1’-3’). The outer borders of *The Rat Brain in Stereotaxic Coordinates* SNR are very similar to the *WHS rat brain atlas* SN-r (Fig. 11a1’-3’,a1”-3”). The part of the *WHS rat brain atlas* SN-r (see above) made up by the SNCV is so small that the SNR in the two atlases can be considered identical.

For the substantia nigra, lateral part (SNL; Fig. 11a1”-3”) in *The Rat Brain in Stereotaxic Coordinates*, 73% is situated within the SN-1 of the *WHS rat brain atlas*. Of the remaining SNL, 12% and 11% is situated within the corticofugal tract and corona radiata and brainstem, unspecified, respectively (Fig. 11b1’-3’). Qualitatively, they appear virtually identical, except that the SNL seems to extend further rostrally in *The Rat Brain in Stereotaxic Coordinates*. In contrast, the entire SN complex extends more caudally in the *WHS rat brain atlas* compared to *The Rat Brain in Stereotaxic Coordinates* (Fig. 11b1-3).

The *Brain Maps* SNc (Fig. 11c1”-3”) is quite different in shape and size compared to the *WHS rat brain atlas* SN-c (Fig. 11c1’-c3’). The SNc in *Brain Maps* is larger in rostral and middle portions (extending dorsolaterally into the *WHS rat brain atlas* SN-l; Fig. 11c2’). Thus, although the *Brain Maps* SNc is mainly situated in the SN-c of *WHS rat brain atlas* (38%), it also has a large portion situated in the SN-r (35%) and the BS-u (14%), and a smaller part in the SN-l (6%) (see Fig. 11d1’-3’). The *WHS rat brain atlas* SN-c, however, has a longer caudal extent. The *Brain Maps* SNc should therefore only be considered to overlap with the *WHS rat brain atlas* SN-c. The *Brain Maps* substantia nigra, reticular part (SNr; Fig. 11c1”-3”) appears relatively similar to the *WHS rat brain atlas* SN-r (Fig. 11c1’-2’), except in the caudal part where it is smaller (Fig. 11c3’,c3”). Quantitatively, it is mainly situated in the *WHS rat brain atlas* SN-r (90%), with smaller portions situated in surrounding regions (see Table 3; Fig. 11d1’-3’).

### Ventral tegmental area

The **ventral tegmental area (VTA**; Fig. 12**)** in *The Rat Brain in Stereotaxic Coordinates* (represented in Fig. 12a,b) consists of five subregions (see Table 1; Fig. 12a1”-3”). Considering the subregions as an overall VTA complex (Fig. 12a1’-3’), the rostral parts of this VTA complex correspond better with the *WHS rat brain atlas* VTA than its caudal parts (Fig. 12a1’,b1’). Generally, the middle and caudal parts of the VTA complex in *The Rat Brain in Stereotaxic Coordinates* extends more dorsally and medially (Figs. 12a2-3, 13a2’-3’), and shows a far larger caudal extent (until the caudal end of the SN; Fig. 12a3’), whereas the *WHS rat brain atlas* VTA only extends about halfway through the SN (Fig. 12a2’).

**Fig. 13:**
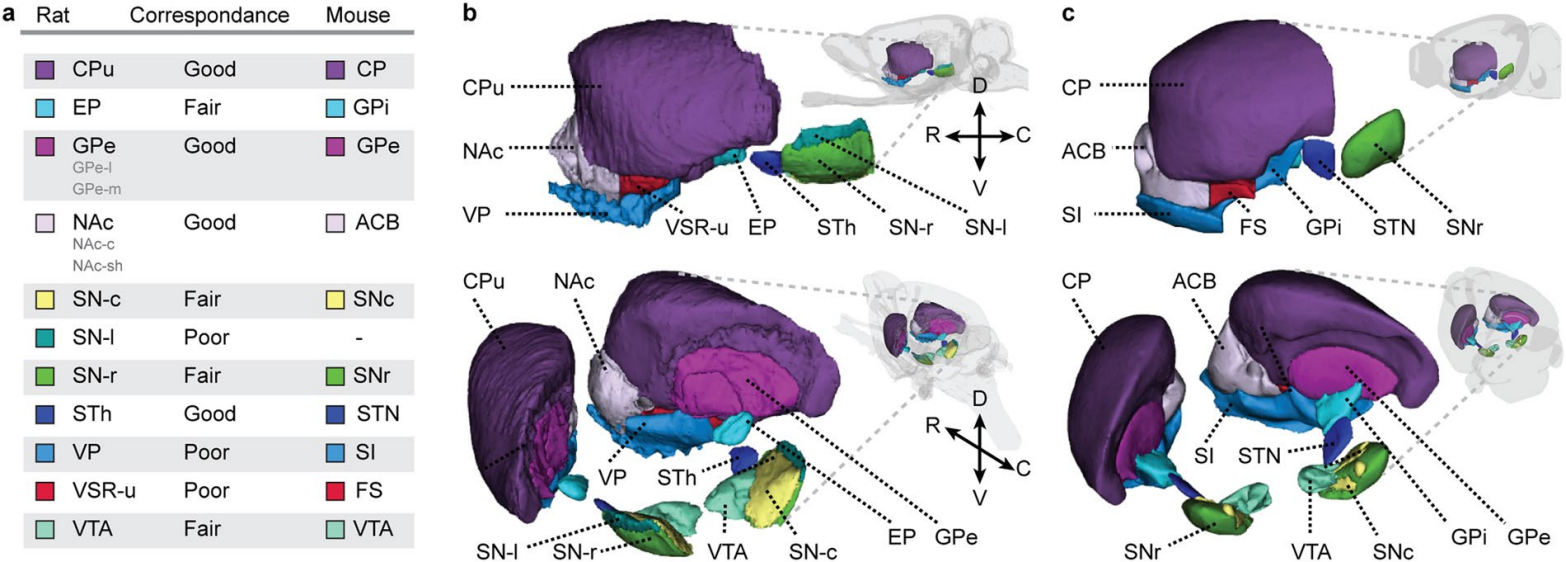
Comparison of basal ganglia regions in the *WHS rat brain atlas v4* and *Allen CCFv3 2017*. (**a**) Overview of the correspondence between regions in the rat and mouse brain atlases, the degree of correspondence was assessed as good, fair, or poor. (**b**) Lateral and caudal views of basal ganglia regions in *WHS rat brain atlas v4*. (**c**) Lateral and caudal views of the corresponding regions in *Allen CCFv3 2017*, colour-coded according to the colour-scheme of the *WHS rat brain atlas v4*. Note that some regions in the *WHS rat brain atlas v4* were merged to maximise correspondence between the atlases (indicated by subregions being listed under the parent region in grey font). **Abbreviations**: ACB, nucleus accumbens; C, caudal; CP, caudoputamen; CPu, caudate putamen; D, dorsal; EP, entopeduncular nucleus; FS, fundus of striatum; GPe, globus pallidus, external segment; GPe-l, globus pallidus external, lateral part; GPe-m, globus pallidus external, medial part; GPi, globus pallidus, internal segment; NAc-c, nucleus accumbens, core; NAc-sh, nucleus accumbens, shell; R, rostral; SI, substantia innominata; SN-c/SNc, substantia nigra, compact part; SN-l, substantia nigra, lateral part; SN-r/SNr, substantia nigra, reticular part; STh/STN, subthalamic nucleus; V, ventral; VP, ventral pallidum; VSR-u, ventral striatal region, unspecified; VTA, ventral tegmental area.

The rostral VTA (VTAR) and parabrachial pigmented nucleus (PBP) in *The Rat Brain in Stereotaxic Coordinates* together correspond to most of the rostral part of the VTA in the *WHS rat brain atlas* (Fig. 12a1”). However, the *WHS rat brain atlas* VTA is more extensive than the combination of the PBP and VTAR rostrally, with a more medial and ventral extent (Fig. 12a1’). The VTAR is almost exclusively situated in the *WHS rat brain atlas* VTA (98%). For the PBP, only about half the region (53%) is situated in the *WHS rat brain atlas* VTA, whereas the remaining is situated in the *WHS rat brain atlas* brainstem, unspecified (43%) (see Fig. 12b1’-3’). The rostral part of the PBP is situated almost exclusively in the *WHS rat brain atlas* VTA (Fig. 12a1’,b1’), but it extends more dorsally and medially in its caudal part (Fig. 12a2’-3’,a2”-3”).

The PN and PIF emerge more caudally in the VTA complex of *The Rat Brain in Stereotaxic Coordinates* (Fig. 12a2”-3”). Both these regions are mainly situated in the *WHS rat brain atlas* brainstem, unspecified (PN: 54%; PIF: 71%) and have less extensive areas situated in the ventral tegmental area (PN: 39%; PIF: 28%) (see Fig. 12b1’-3’). Again, the correspondence is better rostrally: the rostral parts of the PN and PIF are almost fully included in the *WHS rat brain atlas* VTA (Fig. 12a2’,b1’), and the discrepancies appear more caudally (Fig. 12a3’,b2’-3’). The most caudal part of *The Rat Brain in Stereotaxic Coordinates* VTA complex – which is simply termed “VTA” – is situated within the *WHS rat brain atlas* brainstem (~100%; Fig. 12b2’). To summarise, the combined subnuclei of the VTA in *The Rat Brain in Stereotaxic Coordinates* are more extensive than the VTA in the *WHS rat brain atlas* and have a notably larger caudal extent; the exception is the rostral part, where the *WHS rat brain atlas* VTA is slightly larger.

The *Brain Maps* VTA (represented in Fig. 12c,d) is situated partly in the *WHS rat brain atlas* VTA (46%) and partly in the brainstem, unspecified (51%) (see Fig. 12d1’-3’). It has the same rostral extent as the *WHS rat brain atlas* VTA, but the *Brain Maps* VTA is bigger rostrally and has a medial protrusion that is not seen in the *WHS rat brain atlas* (Fig. 12c1’). In the mid-levels of the regions, the VTA in *Brain Maps* and *WHS rat brain atlas* generally appear similar. However, the dorsal border of *Brain Maps* VTA is quite variably defined, with one section showing a large dorsal protrusion of the VTA (Fig. 12c2’), which due to the limited number of sections has a large effect on the quantitative data (Fig. 12d2’). As with the VTA subregions in *The Rat Brain in Stereotaxic Coordinates*, the *Brain Maps* VTA extends more caudal than the *WHS rat brain atlas* VTA, towards the caudal end of the SN (Fig. 12c3’,d3’).

### Atlas metadata organised in openMINDS

The results from the qualitative and quantitative comparison of *Brain Maps* (version 3) and *The Rat Brain in Stereotaxic Coordinates* (version 6) to the *WHS rat brain atlas* (version 4) was represented using the openMINDS SANDS specification, facilitating the use of atlases and regions to integrate data in neuroscience graph databases. Each brain atlas (*WHS rat brain atlas, The Rat Brain in Stereotaxic Coordinates*, and *Brain Maps*) is represented with a schema for the brain atlas, coordinate system, and terminology. Each of these elements have schemas corresponding to specific versions of the brain atlas, i.e. brain atlas version, coordinate system version, and terminology version. Each brain region is represented through a ParcellationEntity and ParcellationEntityVersion schema, which in turn are part of the terminology and terminology version, respectively. In total, this yielded 1159 parcellationEntityVersions for *Brain Maps* (version 3; https://github.com/openMetadataInitiative/openMINDS_instances/tree/main/instances/latest/parcellationEntityVersions/SwansonBM_3rd-ed), 952 for *The Rat Brain in Stereotaxic Coordinates* (version 6; https://github.com/openMetadataInitiative/openMINDS_instances/tree/main/instances/latest/parcellationEntityVersions/PW-RBSC-cor_6th-ed), and 222 for the *WHS rat brain atlas* (version 4; https://github.com/openMetadataInitiative/openMINDS_instances/tree/main/instances/latest/parcellationEntityVersions/WHSSDatlas_v4). Quantitative and qualitative relationships between regions are represented through quantitativeAssessment and qualitativeAssessment schemas, respectively. In total, we registered 243 quantitative relationships and 46 qualitative relationships from the analysis presented here. The region relations were added to the atlas metadata in the EBRAINS Knowledge Graph (KG) and can be access via its REST-API (https://core.kg.ebrains.eu). Taken together, openMINDS provides a library of atlas metadata instances that can be used in graph databases such as the EBRAINS KG. This ensures that the metadata can be queried and used in other research or in combination with other data from the EBRAINS KG.

### Comparison of rat and mouse basal ganglia regions

To provide a basis for cross-species comparison of data from basal ganglia regions, we compared the basal ganglia in the *WHS rat brain atlas v4* to those in the *Allen Common Coordinate Framework version 3, 2017* (*Allen CCFv3 2017*). In general, the *WHS rat brain atlas v4* has a higher level of detail, with more subdivisions, including e.g. annotations of the NAc core and the GP medial and lateral parts. For cross-species comparisons, such differences can be resolved by merging regions in the *WHS rat brain atlas v4* for analysis (Fig. 13b,c).

Compared qualitatively, with respect to shapes and relative sizes of regions, the CP, NAc/ACB, GPe and STh/STN show a high level of correspondence between the two atlases (Fig. 13a). A few differences can be highlighted. The VSR-u in the *WHS rat brain atlas v4* (Fig. 13b) does not have a directly corresponding region in the *Allen CCFv3 2017* but based on its shape and location most closely corresponds to the FS (Fig. 13c). The GPi in the *Allen CCFv3 2017* has a reasonable correspondence to the EP of the *WHS rat brain atlas v4*, but also resembles the region with the same term in the Swanson rat brain atlas v3, being partly situated in the internal segment and partly encapsulated in the peduncle. The VP does not exist in the *Allen CCFv3 2017*. The closest corresponding region would be the SI, which is far more extensive in the *Allen CCFv3 2017* than the VP in the *WHS rat brain atlas*, extending further caudally into areas that are part of the basal forebrain in *WHS rat brain atlas*. In the *Allen CCFv3 2017* the SN is subdivided into a reticular and compact part, whereas the lateral part only exists in the ontology and not as an annotation. The area that would have corresponded to the SN-l of the *WHS rat brain atlas v4* is situated partly in the brainstem and partly in the SN-r of the *Allen CCFv3 2017*. This is based on the observation that the *Allen CCFv3 2017* SN does not appear to extend as far dorsolateral as the *WHS rat brain atlas v4* SN. The VTA seems to be slightly more extensive in the *WHS rat brain atlas v4* than in the *Allen CCFv3 2017*.

## Discussion

The basal ganglia regions have long attracted interest by neuroscientists for their involvement in motor and cognitive functions, but the terms and boundaries for each region are debated and have changed over time. To aid researchers in understanding how these are defined across murine atlases, we have here presented an overview of the terms and boundaries of basal ganglia regions across atlases and the literature. In the following, we first discuss how terms and boundaries change across atlases and versions, highlighting some of the challenges such changes pose and how our quantitative and qualitative methods of comparing region annotations can mitigate some of these. We also discuss how organising the atlas information in a standardised metadata framework can facilitate integration of data in neuroscience databases.

In our investigation of different atlases and their versions, we observed that terms and boundaries for some regions have changed frequently and substantially. Large differences across atlases or their versions may result from new knowledge, use of different expertise, or possibly reflect controversies in the field. For example, the ongoing debate about homologies and the resulting proper naming for pallidal subregions^40^ is likely to explain the substantial differences in boundaries and frequent modifications in nomenclature for pallidal regions seen across atlases. In some atlas versions, terms have changed without any modification of their boundaries, e.g. the change from “internal globus pallidus (intrapeduncular nucleus)” to “entopeduncular nucleus” between versions 5 and 6 of *The Rat Brain in Stereotaxic Coordinates*^18,19^. In other instances, large changes have been introduced to both terms and boundaries, e.g. between versions 6 and 7 of *The Rat Brain in Stereotaxic Coordinates*, where the previously termed “globus pallidus” was subdivided into an external and internal segment while the previous “internal globus pallidus” was relabelled as “entopeduncular nucleus”.

Another reason for the variability in region boundaries may be that boundaries within and between basal ganglia regions are notoriously difficult to define, with many areas separated by transitional zones rather than sharp boundaries. For example, the rostrodorsal part of the nucleus accumbens shell has been proposed to constitute a basal forebrain transitional area, displaying projection patterns that resemble the dorsolaterally adjacent lateral septum^71^. We also found that the zone of transition between caudal parts of the nucleus accumbens and other basal ganglia and basal forebrain regions was very variable across atlases and difficult to parse in the Waxholm Space rat brain atlas; this area is indeed considered a transition area towards the extended amygdala^33^. Regardless of the reasons for changing atlas terms or boundaries, it is problematic that such changes are poorly described, or often not even mentioned, making it necessary to inspect atlas plates or lists of structures from two versions side-by-side to detect the introduced changes. This points to the need for change-logs specifying how versions of the same brain atlas are modified^72^.

Our spatial comparisons of basal ganglia regions across atlases highlights the challenge of having multiple alternative nomenclatures and definitions for these regions, sometimes referred to as the “brain atlas concordance problem”^73^. While the use of different terminologies certainly contributes to this challenge, it is clear from our results that differences between atlases are not restricted to the use of alternative terms for similarly defined regions. On the contrary, our results demonstrate that for the rat basal ganglia, boundaries vary considerably across atlases and their versions. This corroborates earlier findings from the human thalamus and the rodent prefrontal cortex^74,75^, where substantial differences were seen when different atlases were consulted or even when different experts annotated the same data. While some authors have focused on unifying regions from different atlases^76^, there are already a wealth of available atlas versions in use, and it is unrealistic to expect a single one to become the standard. Our concepts of the basal ganglia regions and their boundaries are likely to evolve further due to methodological advances for mapping cell populations. Rather than seeking a definitive consensus on the definition of basal ganglia regions, the current paper provides the means to interpret, integrate and compare experimental data analysed using different atlases.

Our study was based on performing spatial comparisons of regions in co-registered brain atlases. Both the qualitative and quantitative aspects of such analyses yield important and different insights. Quantitative information about the overlap of a given region with any region in the atlas used as a reference may, e.g., reveal that 40% of region A in atlas X is situated in region B of atlas Y. However, it does not reveal *where* region A overlaps with region B. While this may be partially resolved by splitting the analysis into smaller parts (such as the rostro-caudal segments used in this study), this does not replace a visual qualitative inspection, which gives a better understanding of the overlap in-plane and in relation to surrounding regions.

When observing qualitative or quantitative differences between regions across atlases, multiple explanations must be considered. First, because we are comparing atlases based on animals of different strain and age, differences between regions across atlases can also be influenced by inter-animal differences. In addition, differences can be introduced by variability in deformations caused by tissue processing^77^. Secondly, 2D stereotaxic atlases are based on systematically sampled sections from the brain; thus, our results pertain to the sampled proportion of sections and available material for each atlas (see Methods for details). While these should theoretically provide a representative sample of the entire region, it is possible that the results would be different if more sections were included. Thirdly, an important factor is the methods used to spatially register atlas plates from 2D atlases to the 3D Waxholm Space rat brain atlas. Any inaccuracies in this registration will influence the comparative analysis. To ensure the highest quality possible, our registrations were assessed and agreed upon by two investigators, and non-linear refinement was implemented to allow quantitative analysis and to minimise the effect of differences in animals and tissue processing. Lastly, differences may result from differences in delineation criteria; these are the differences we aim to capture in the analyses. Thus, interpreting differences between atlas region boundaries is a complex task, where the qualitative and quantitative methods have important and complementary value.

In some cases, our qualitative and quantitative analyses yielded different results. For example, in the case of the subthalamic nucleus, differences that were judged to be minimal in the qualitative assessment but showed relatively large discrepancies quantitatively. One interpretation is that discrepancies might be underestimated in a qualitative analysis. However, the low correspondence in the quantitative analysis is likely also an effect of the size of the region. For small regions, small discrepancies may have large effects on the quantitative data and they are likely to be disproportionally affected by sampling in the stereotaxic atlases as well as variability between animals and tissue processing procedures. These considerations can be accounted for in a qualitative inspection.

All the results from our qualitative and quantitative comparison analyses are shared openly on the EBRAINS Knowledge Graph, and we registered atlas metadata and relationships between regions in the openMINDS metadata framework. This standardises the results from our study for use in neuroscience graph databases and increases comparability of the different atlases. Currently, the atlas metadata can be found both in the openMINDS GitHub repository and the EBRAINS KG, while region relations can be found in the EBRAINS Knowledge Graph. All metadata can be queried programmatically, in accordance with the FAIR principles^78^. Future efforts should investigate how such detailed metadata can be utilised and presented in neuroscience databases to improve data integration and interoperability. For example, the relation assessments could be used to find data coupled to the closest matching region(s) in different atlases, with the quantitative and qualitative assessments available to aid the user in understanding potential differences between the datasets. They could further aid as a change-log between versions of the same atlas and increase interoperability between different atlases by structuring relevant metadata in a standardised manner.

To summarise, we have provided an extensive overview of terms and boundaries used for basal ganglia regions in the murine literature and rat brain atlases. We hope this will provide researchers with a better understanding of the various concepts, boundaries, and terminologies. Our analysis was performed with open-source tools and our data and code is publicly available, paving the way for similar analyses of other regions. While the tools used are currently specialised for handling murine brain data, we believe the principles behind our analysis are broadly applicable across species. We hope that this will motivate further investigations into the variability of brain region terms and boundaries across atlases, ultimately equipping researchers with the knowledge needed to integrate and compare data across neuroscientific studies.

## Methods

### Definition of the basal ganglia

We here consider the rat and mouse (collectively referred to as murine) basal ganglia to include the following regions: the caudate-putamen, nucleus accumbens, and ventral striatum (collectively referred to as the striatum); the globus pallidus, ventral pallidum, and entopeduncular nucleus (collectively referred to as the pallidum); the subthalamic nucleus; the substantia nigra; and ventral tegmental area. Whether the entopeduncular nucleus is part of the murine basal ganglia is a matter of debate (see “Overview of basal ganglia regions, nomenclature and hierarchy” in the Results section), but we choose to include it as part of the basal ganglia given its long history of being considered the murine homologue of the human internal globus pallidus^5^.

### Delineating basal ganglia in the waxholm space atlas of the sprague dawley rat brain

The basal ganglia regions of the *Waxholm Space atlas of the Sprague Dawley rat brain* (*WHS rat brain atlas*; RRID:SCR_017124)^9,79–81^ were delineated using the following files from the atlas home page on NITRC (www.nitrc.org/projects/whs-sd-atlas):

- **WHS_SD_rat_T2star_v1.01.nii.gz:** Structural magnetic resonance image (sMRI) acquired as *T*_2_^*^- weighted gradient recalled echo (GRE) images, at 39 μm isotropic resolution (1024x512x512 voxels). See^79^ for technical details about MRI acquisition.
- **WHS_SD_rat_DWI_v1.01.nii.gz:** Diffusion weighted imaging (DWI) maps, acquired with diffusion sensitisation along six non-colinear diffusion gradient vectors at 78 µm isotropic resolution, resampled to 39 µm resolution to match the sMRI data (1024x512x512 voxels). See^79^ for technical details about MRI acquisition.
- **WHS_SD_rat_FA_color_v1.01.nii.gz:** Diffusion tensor image (DTI) maps derived from DWI data, showing Fractional Anisotropy (FA) values computed from three principal eigenvalues after tensor decomposition, displayed as red-green-blue (RGB) maps of principal eigenvector orientation with FA shown as image intensity, resampled to 39 μm resolution (1024x512x512 voxels). See^79^ for technical details about MRI acquisition.
- **WHS_SD_rat_atlas_v3.nii.gz:** Volumetric atlas file containing 118 annotations.
- **WHS_SD_rat_atlas_v3.label:** File specifying the ID, colour-code, and term for each annotation.

**Fig. 14:**
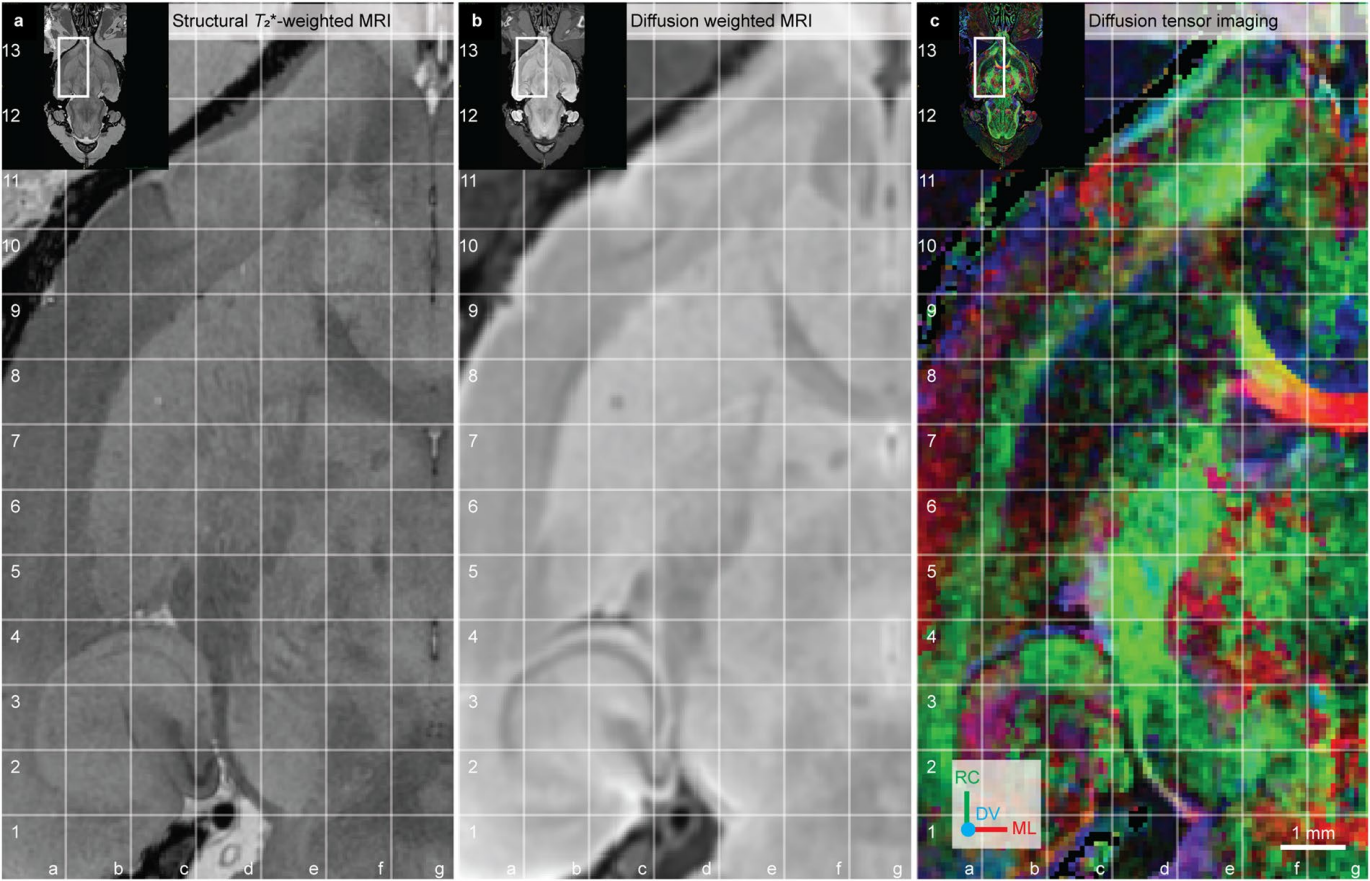
The *WHS rat brain atlas* reference data. The *WHS rat brain atlas* annotations are based on a multimodal magnetic resonance imaging (MRI) dataset consisting of (**a**) structural *T*_2_*-weighted MRI maps, (**b**) diffusion weighted MRI maps, and (**c**) diffusion tensor imaging maps, with DTI orientation colour code inset in (**c**) **Abbreviations:** DV, dorsoventral; ML, mediolateral; RC, rostrocaudal.

The procedure for delineating brain regions has been described extensively in previous publications^9,79–81^. Briefly, the reference dataset is interpreted and manually annotated in 3D using the ITK-SNAP software^82^. The reference dataset consists of a structural magnetic resonance image (sMRI, Fig. 14a), a diffusion weighted image (DWI, Fig. 14b) and a diffusion tensor image (DTI, Fig. 14c) which each reveal different brain area characteristics. In the sMRI maps, the cerebral cortex appears with relatively lower signal intensity (dark-grey colour; Fig. 14, grid b10) compared to the caudate-putamen appearing as an area with higher signal intensity (brighter grey; Fig. 14a, grid c8), while white matter fibre tracts generally appear as areas with relatively low signal intensity (darker grey; Fig. 14, grid g8). Similar relative differences are seen in the DWI maps (compare Fig. 14b, grid b10, c8 and g8). The tissue appears more homogenous in DWI compared to sMRI. In DTI, the voxel colours represent the orientation of the principal eigenvector, which reflects the average direction of water diffusion in a voxel. The principal orthogonal directions have a red-green-blue (RGB) colour-code: red-mediolateral, green-rostrocaudal, and blue-dorsoventral. Areas with highly oriented water diffusion, such as white matter fibre tracts, are visible in the DTI maps as brightly coloured voxels (high FA signal intensity; see corpus callosum in Fig. 14c, grid g8). In cell-rich grey matter areas, the direction of water diffusion is generally less oriented, visible as low signal intensity and less bright colours in DTI maps (low FA values; see cortex in Fig. 14c, grid b10). Areas with a mix of cells and fibres, display heterogeneous average orientations, visible as a mixture of colours in DTI maps (Fig. 14c, e.g. caudate-putamen in grids c7 and c8, or cerebral cortex in grid b10). To aid the interpretation of the reference dataset, we used existing collections of histological material, including brain sections stained using thionine and Woelche’s myelin staining method^83^.

### Investigating basal ganglia regions across rat brain atlases

To give an overview of how basal ganglia regions vary and relate across rat brain atlases and their versions, we surveyed the terms and annotations of basal ganglia regions across versions of the most commonly used stereotaxic atlases. To provide a basis for comparing annotations across atlases, we spatially registered atlas images from these stereotaxic atlases to the *WHS rat brain atlas*. Based on this, we performed quantitative and qualitative comparisons of the spatial relationship of regions in stereotaxic atlases with regions in the *WHS rat brain atlas*. In the following, we first give a brief overview of the surveyed atlases, before describing the methodological details for each of the steps listed above.

### Surveyed atlases

We focused our surveys and analyses on the most commonly used stereotaxic rat brain atlases, i.e. *The Rat Brain in Stereotaxic Coordinates* by Paxinos and Watson^10,12,15–19^ and *Brain Maps: Structure of the Rat Brain* by Swanson^11,13,20,21^. Both these atlases are based on adult male rat brains (as is the *WHS rat brain atlas*), and include detailed delineations covering the entire brain; however, the level of detail varies across atlases and their versions. A more detailed overview of the reference data used in all the atlases surveyed here can be found in Table 5.

**Table 5:** Overview of atlases surveyed in this study.

*Rat brain atlases*						
*Atlas*	Reference data	Displayed planes	Sex	Strain	Age	Weight
*WHS rat brain atlas v4*	3D MRI/DTI	—	Male	Sprague Dawley	80 days	397.6 g
*The Rat Brain in Stereotaxic Coordinates v1–4*	2D histology	Coronal	Male	Wistar	Adult	300 g
	2D histology	Sagittal				270 g
	2D histology	Horizontal				290 g
*The Rat Brain in Stereotaxic Coordinates v5–7*	2D histology	Coronal	Male	Wistar	Adult	290 g
	2D histology	Sagittal				270 g
	2D histology	Horizontal				290 g
*Brain Maps v1–4*	2D histology	Coronal	Male	Sprague Dawley	80 days	315 g

In this study, we compared basal ganglia regions across *The Rat Brain in Stereotaxic Coordinates* by Paxinos and colleagues and from *Brain Maps* by Swanson to *WHS rat brain atlas v4*. Details about the subjects used for the reference data in the various atlases are listed in the table.

*The Rat Brain in Stereotaxic Coordinates* was first published in 1982 and currently has seven versions. Some of the atlases have also been published as both full and compact versions, where the compact versions only contain the coronal reference images and annotated plates. In our investigations, we used the full versions for version 1–2 and 4–6, and the compact version 3 and 7. While the full versions include annotated sections from all three planes, we only considered the coronal sections as this series is most comprehensive. All the surveys and analyses described in the following therefore exclusively use the coronal material provided in *The Rat Brain in Stereotaxic Coordinates*. The reference datasets used in *The Rat Brain in Stereotaxic Coordinates* are from the adult male, Wistar rat brain. The first four versions use the same brain sample as the reference, but this dataset was of limited section sampling frequency, and some damaged sections had been replaced with corresponding sections from a different brain^18^. The number of sections were substantially increased between the first two versions, from 43 to 76 annotated plates, while versions 3^16^ and 4^17^ include 78 coronal sections. For version 5^18^, a new brain was acquired for the coronal reference dataset, such that version 5–7 features 161 coronal sections from a single brain. All versions of *The Rat Brain in Stereotaxic Coordinates* are copyrighted by Elsevier.

*Brain Maps: Structure of the Rat Brain* was first published in 1992 and currently has four versions. The atlas features 73 coronal diagrams from an adult male Sprague Dawley rat, and the reference dataset is the same across all versions. The annotations are drawn on one side of the brain only, and in some versions flipped to create synthetic bilateral annotations. The atlases were recently made openly available under a CC-BY-NC licence.

### Survey of naming conventions and annotations across stereotaxic atlas versions

We first compared the terms and annotations for basal ganglia or closely related regions across versions 1–7 of *The Rat Brain in Stereotaxic Coordinates*^10,12,15–19^ and versions 1–4 of *Brain Maps: Structure of the Rat Brain*^11,13,20,21^. We used the index of abbreviations in the respective atlases. We only considered regions that appeared both in the index of abbreviations *and* as an annotation in the serial diagram images (hereafter referred to as atlas plates). In some cases, region terms were followed by the name of the person who first named it (e.g. “subthalamic nucleus (Luys)”). We did not consider such names in brackets to be part of the region term.

Changes in annotations across versions were inspected in a side-by-side comparison of different versions of the atlases. For a given region in each version, we classified the “Status” as “unchanged”, “minor changes”, “major changes,” “new”, or “replaced”. A region was only considered unchanged if there were no visibly detectable change in the annotation in any atlas plate; in cases where new plates were added (such as between version 1 and 2 of *The Rat Brain in Stereotaxic Coordinates*), we did consider the region unchanged if there were no changes to the previously included sections. We considered a region to have undergone minor changes if there were any change at all in its borders; more substantial changes to a region’s shape, size or rostrocaudal extent were considered to constitute major changes. Regions that were not present in a previous version were given the status “new”.

### Spatial registration of atlas plates to *WHS rat brain atlas v4*

We previously performed spatial registration of the atlas plates from the stereotaxic atlases to the *WHS rat brain atlas v4*^60^, using the QuickNII software for registration of 2D section images to 3D atlases (RRID:SCR_016854; https://quicknii.readthedocs.io)^84^. Coronal diagram images from each atlas were used in the registration, and deviations from the sectioning plane were determined by inspecting the relationship between landmarks in dorsal versus ventral (dorsoventral angle) or left versus right (mediolateral angle) parts of the section. The registration parameters (angles and positions of the sections) were kept identical for atlas versions with the same reference data. The.xml files (compatible with QuickNII) defining the registration of each atlas to the *WHS rat brain atlas v4* are shared through the EBRAINS Knowledge Graph (RRID:SCR_017612)^69,85–93^.

For the current analysis, we refined the published registrations using the VisuAlign tool (RRID:SCR_017978; https://visualign.readthedocs.io)^94^, which allows in-plane nonlinear refinements of spatially registered images. In doing so, we were cautious to implement only minimal adjustments that would account for plausible differences between animals used in the reference data of the respective atlases and for deformities caused by processing and mounting of the histological atlases, while not masking actual differences in annotations. The resulting custom-cut and non-linearly refined atlas plates were exported and used in the quantitative analysis (see below).

### Qualitative analysis of overlap between atlas annotations

To compare basal ganglia regions across atlases, we inspected images from each atlas with the corresponding *WHS rat brain atlas v4* overlay, toggling the overlay to assess the brain region correspondence. For any region in the compared atlases that showed some relationship with a basal ganglia region in the *WHS rat brain atlas v4*, we noted the type of relationship, defined as identical, part of, including, overlapping, or non-overlapping. “Non-overlapping” was only used in instances where two regions shared the same term but did not spatially overlap. We also used a region comparability score to semi-quantitatively assess the similarity of two regions and wrote detailed comments about the relationship. The qualitative comparison workflow has been described more extensively in our previous study^60^. In cases where annotations were unchanged across versions of a reference atlas, we only assessed the last version and noted down the other versions for which the assessment were valid.

### Quantitative analysis of overlap between atlas annotations

To expand upon our qualitative descriptions of the relationships between atlas annotations, we performed a quantitative analysis of the spatially registered annotations for one version of *The Rat Brain in Stereotaxic Coordinates* (v6)^19^ and one version of *Brain Maps* (v3)^21^. To perform quantification, we created versions of the atlas plates with basal ganglia regions redrawn and colour coded. For each region, the coloured pixels in each atlas plate and their overlap with regions in *WHS rat brain atlas v4* was quantified using Nutil quantifier (v0.8.0; RRID:SCR_017183; https://nutil.readthedocs.io)^95^. For this analysis, object splitting was turned on, and all other parameters followed the default values.

Nutil quantifier takes as input a set of serially ordered section images. In this case, the input images are atlas plates from *The Rat Brain in Stereotaxic Coordinates* (v6)^19^ or *Brain Maps* (v3)^21^, where the annotations for all basal ganglia region has been redrawn. Each input image is analysed by a custom-cut atlas plate from the selected atlas (in this case, the *WHS rat brain atlas v4*), as exported from VisuAlign (see above). The result is a set of section-wise reports, where each report gives the number of object pixels in regions of the *WHS rat brain atlas v4* atlas. Thus, in this case, the object corresponds to a basal ganglia region in *The Rat Brain in Stereotaxic Coordinates* (v6)^19^ or *Brain Maps* (v3)^21^, and the object pixels represent the number of pixels from that region that is situated in a given region in the *WHS rat brain atlas v4*. For further analysis, we compiled all the section-wise data into a single report with the section number as a column. For each region in each section, we calculated the overlap of a region with *WHS rat brain atlas v4* regions as follows:

$$\frac{{{OP}}_{{reg}}}{\sum {OPs}}$$

Where OP_reg_ is the number of object pixels in a region of *WHS rat brain atlas v4* and ∑OP_s_ is the sum of object pixels in the section. This gives the section-wise proportion of a region in an atlas (e.g. the nucleus accumbens shell in *The Rat Brain in Stereotaxic Coordinates*) that is situated in a given region of the *WHS rat brain atlas v4*.

We grouped the *section*-wise data into rostral, middle, and caudal segments. If the number of sections were not divisible by three, we allocated the rest to the middle segment. We then calculated the proportion of the region in each *WHS rat brain atlas v4* region in the same way as for the section-wise data (see above) and created pie charts of the proportion of different atlas regions in *WHS rat brain atlas v4* per segment. Lastly, we also calculated the proportion of overlap for each region across all segments and sections. All calculations and graphing were performed in Python (version 3) using custom code available at https://github.com/ingvildeb/basal_ganglia_project.

### Organisation and sharing of data

We organised and indexed atlas metadata to ensure the information on how basal ganglia regions vary across rat brain atlases and their versions is as accessible as possible, considering both human- and machine readability. To record the results systematically, we first designed a relational database to store information about atlases, regions, and their relationships.

### Database of basal ganglia region terms and relationships across atlases

To record the results of the atlas investigations described above systematically, we designed a relational database in Microsoft Access to store information about atlases, regions, and their relationships. The database has a simple design with three tables (“Nomenclatures”, “Regions”, and “Topological_relations”). The nomenclature table contains information about each atlas version, including publication year and information about the reference data version. The region table is linked to the nomenclature table and contains information about the region term, abbreviation, Bregma level of the rostral and caudal end, number of sections, and information about potential changes from the previous version. Lastly, the topological relations table links two regions from the regions table and contains information about their topological relationship with detailed comments. The database, and detailed documentation of all tables, fields, and relations, is available from the EBRAINS Knowledge Graph^67^.

### Representation of atlas metadata and region relationships in openMINDS

To facilitate the use and machine-readability of atlas elements and the integration of data that uses different atlases, we represented the results from this study (qualitative and quantitative relationships) in openMINDS (open Metadata Initiative for Neuroscience Data Structures; RRID:SCR_023173), a metadata framework for graph databases adopted in the EBRAINS Knowledge Graph. We focused this effort on the versions used in both the quantitative and qualitative analyses.

To represent the relationships between regions across atlases, it was first necessary to represent the metadata of the atlases and regions for each atlas. Atlas metadata include term, version and licence, as well as descriptions and definitions of the coordinate system, reference data, terminology and annotations^72^. We collected the atlas metadata for all versions of the *WHS rat brain atlas* and one version each of *The Rat Brain in Stereotaxic Coordinates* (version 6) and *Brain Maps* (version 3) following the openMINDS SANDS specification (Spatial Anchoring of Neuroscience Data Structures; RRID:SCR_023498). In SANDS, atlases are captured as a combination of metadata schemas representing the components mentioned above. For detailed information about the schemas, see the “atlas schema specifications” on the openMINDS webpage (https://openminds-documentation.readthedocs.io/en/v3.0/schema_specifications/SANDS/atlas.html). Briefly, atlases are represented by schemas for BrainAtlas, BrainAtlasVersion, CommonCoordinateSpace, CommonCoordinateSpaceVersion, ParcellationTerminology, ParcellationTerminologyVersion, ParcellationEntity, ParcellationEntityVersion and AtlasAnnotation. We registered each atlas as a BrainAtlasVersion with a CommonCoordinateSpaceVersion, the coordinate system used in the atlas, and a ParcellationTerminologyVersion. The terminology contains ParcellationEntityVersions with the corresponding AtlasAnnotation representing the regions in the atlas. Because the AtlasAnnotation should preferably be linked to a file representing the annotation, we attached such schemas for the *WHS rat brain atlas* only, as it comes with a digital file for the annotations. All versions of an atlas are grouped under the BrainAtlas schema and all coordinate system versions are group under the CommonCoordinateSpace schema. The BrainAtlas also has a ParcellationTerminology that contains the conceptual ParcellationEntities that are used across all versions of the atlas.

Next, qualitative and quantitative relationships were represented through the SANDS schemas qualitativeRelationAssessment and quantitativeRelationAssessment (https://openminds-documentation.readthedocs.io/en/v3.0/schema_specifications/SANDS/miscellaneous.html). These schemas are embedded on a ParcellationEntityVersion, and define the criteria used to assess the relation as well as a link to the ParcellationEntityVersion to which the relation is described. In the qualitativeRelationAssessment schema, the qualitative relationship is given as “isIdenticalTo”, “hasIntersectionWith”, “isSubsetOf” or “isSupersetOf”, corresponding to the relationships termed “identical”, “overlapping”, “part of” and “includes” in the relational database^67^. In this source database, *part of* and *includes* relationships were only represented in one direction (e.g. the *Brain Maps v3* nucleus accumbens **includes** the *WHS rat brain atlas v4* nucleus accumbens, core. When representing these relationships in openMINDS, we also represented the opposite relation (the *WHS rat brain atlas v4* nucleus accumbens, core **is part of** the *Brain Maps v3* nucleus accumbens). In the quantitativeRelationAssessment schema, the quantitative overlap is given as a percentage. These percentages were extracted from the summary files for each atlas (shared with the corresponding dataset)^70^. Quantitative relationships were only represented for regions where the quantitative overlap with a region in the *WHS rat brain atlas* was equal to or more than 0.01%.

### Comparative analysis of rat and mouse basal ganglia

To facilitate cross-species comparisons, we compared the basal ganglia regions in the *Allen Mouse Brain Common Coordinate Framework* (version 3, 2017 edition of the annotations; *Allen CCFv3 2017*; RRID:SCR_020999)^14^ to those in the *WHS rat brain atlas v4*. An overview of the regions in *Allen CCFv3 2017* here considered as part of the basal ganglia, as well as nomenclature changes across annotation editions, is included in Table 6.

**Table 6:** The basal ganglia in the *Allen Mouse Brain atlas Common Coordinate Framework version 3*.

Basal ganglia in the *Allen Mouse Brain atlas Common Coordinate Framework* version 3		
Abbreviation	Term	Edition specification
GPe	Globus pallidus, external segment	2015; 2016; 2017
GPi	Globus pallidus, internal segment	2015; 2016; 2017
SI	Substantia innominata	2017
STR	Striatum	2015; 2016; 2017
CP	Caudoputamen	2015; 2016; 2017
FS	Fundus of striatum	2017
ACB	Nucleus accumbens	2015; 2016; 2017
STN	Subthalamic nucleus	2015; 2016; 2017
SNc	Substantia nigra, compact part	2017
SNl	Substantia nigra, lateral part	*
SNr	Substantia nigra, reticular part	2015; 2016; 2017
VTA	Ventral tegmental area	2017

Based on the Allen Brain Atlas (ABA) Adult Mouse Brain Ontology (https://bioportal.bioontology.org/ontologies/ABA-AMB) and white papers from 2015, 2016 and 2017. *The SNl is included in the nomenclature, but not provided as an annotation.

We qualitatively assessed the spatial correspondence of each region pair and scored them using a three-step rating scale (good, fair, or poor), based on visual inspection of the regions in the MeshView tool (RRID:SCR_017222; https://meshview-for-brain-atlases.readthedocs.io) for the Waxholm Space atlas of the Sprague Dawley rat brain v4 (v0.8 h; https://meshview.apps.hbp.eu/?atlas=WHS_SD_Rat_v4_39um) and Allen Mouse Brain Atlas CCFv3 2017 (v0.8 h; https://meshview.apps.hbp.eu/?atlas=ABA_Mouse_CCFv3_2017_25um). This assessment considered the shape of the region and its extent relative to surrounding areas. To facilitate the visual comparisons, the atlases were displayed side-by-side, and the colour-codes of the *Allen CCFv3 2017* were manually adjusted to correspond to the colour-scheme used in the *WHS rat brain atlas v4*. Some of the basal ganglia annotations in the *WHS rat brain atlas v4* are more fine-grained than in the *Allen CCFv3 2017*; in these cases, we considered the correspondence of the *Allen CCFv3 2017* region with the merged version of relevant regions in the *WHS rat brain atlas v4*.

## Data Availability

All the data from this study is available from the EBRAINS Knowledge Graph: https://search.kg.ebrains.eu. The spatial co-registration datasets are available from the project titled “Spatial relationships among murine brain atlas coordinate systems“^69,85–93^. The data from the quantitative spatial correspondence analysis is available from the dataset titled “Quantitative comparison of basal ganglia delineations across different rat brain atlases (v1)”^70^. The data from the qualitative spatial correspondence analysis is available from the dataset titled “Comparability of basal ganglia delineations across different rat brain atlases (v2)”^67^. The Waxholm Space atlas of the Sprague Dawley rat brain version 4 is available from the atlas home page on NITRC (www.nitrc.org/projects/whs-sd-atlas).

## Code availability

All the code used in this study is publicly available via Github: https://github.com/ingvildeb/basal_ganglia_project.
